# The many facets of shape

**DOI:** 10.1167/jov.22.1.1

**Published:** 2022-01-04

**Authors:** James T. Todd, Alexander A. Petrov

**Affiliations:** 1 Department of Psychology, The Ohio State University, Columbus, OH, USA; 2 Department of Psychology, The Ohio State University, Columbus, OH, USA

**Keywords:** 3D surface and shape perception, shape and contour, non-Euclidean geometry

## Abstract

Shape is an interesting property of objects because it is used in ordinary discourse in ways that seem to have little connection to how it is typically defined in mathematics. The present article describes how the concept of shape can be grounded within Euclidean and non-Euclidean geometry and also to human perception. It considers the formal methods that have been proposed for measuring the differences among shapes and how the performance of those methods compares with shape difference thresholds of human observers. It discusses how different types of shape change can be perceptually categorized. It also evaluates the specific data structures that have been used to represent shape in models of both human and machine vision, and it reviews the psychophysical evidence about the extent to which those models are consistent with human perception. Based on this review of the literature, we argue that shape is not one thing but rather a collection of many object attributes, some of which are more perceptually salient than others. Because the relative importance of these attributes can be context dependent, there is no obvious single definition of shape that is universally applicable in all situations.

## The many facets of shape

We all use the word “shape” in our day-to-day discourse, but a precise definition of that term is surprisingly difficult to formulate. Consider the following sentences:

1. The three-dimensional (3D) modeling software provides a set of primitive shapes, including a sphere, a cube, a cylinder, and a pyramid, which can all be deformed and/or combined to create an infinite variety of more complex shapes.
2. The old woman lived in a house, whose shape resembled a shoe.
3. All John has left from his boxing career is his misshapen nose.
4. We all recoiled at the grotesque shape of the creature, whose head was covered with small pointed horns and two writhing tentacles on each side.
5. The sculpture was shaped like a geographic surface with hills, dales, valleys, and ridges.

Note that the individual shape primitives described in the first sentence have specific mathematical definitions, but how do we describe the more complex shapes that are derived from them? In the second sentence, the shape of the house is described by comparing it to something else (i.e., a shoe). This is also the case for the third example except that the “something else” is recognized implicitly as the shape of a normal nose. If we cannot describe a shape by comparing it to something else, we often resort to describing it as a configuration of namable parts as in the fourth and fifth sentences.

With the exception of the basic primitives in the first example, all of these shape descriptions are remarkably vague. If we define a shape (or a part of a shape) by its resemblance to some other form with which we are familiar, then we are still left with the problem of defining the shape of that familiar form. This type of language is only meaningful to the extent that we have a shared understanding about the overall shapes of namable objects (or parts).

There are an infinite number of possible physical measures of an object, but how do we decide which ones should be referred to as shape? Should this be decided by fiat from some prominent mathematical authority, or should we trust our own perceptual intuitions? Our approach as we begin this discussion is to keep an open mind about what object properties constitute shape. We will assume, however, that any theory of shape must ultimately be grounded in human perception. Thus, if two shapes are similar by a mathematically defined measure of shape, they should also be perceptually similar as well.

The present article is divided into two parts: The first part will focus on the measurement of shape differences. It will consider how the congruence and similarity of objects are established within classical geometry. It will also discuss the correspondence relations between objects and how different types of shape change can be categorized. The second part will consider a variety of data structures for the representation of shape, and it will also propose a set of criteria for evaluating those as possible models of human perception. Based on this review of the literature, we will argue that shape should be considered as a collection of many object attributes, some of which are more perceptually salient than others (see also Green, 2017; Koenderink, 1990). This suggests that there may not be a single definition of shape that is wholly satisfactory, because different sets of attributes may be relevant in different contexts (Kimia, Tannenbaum, & Zucker, 1995). For example, the metric properties of Euclidean geometry may be of paramount importance to a tool and die maker but not so much to a biologist who is trying to classify the biological forms of different species.

## Methods of shape comparison

### Shape-related concepts in classical geometry

Classical geometry provides precise formal definitions for a relatively small number of basic shapes. For example, a sphere is defined as the locus of points in three-dimensional (3D) space that are all equidistant from a central point. Similarly, a square is defined as a polygon with four sides of equal length whose adjacent sides are all orthogonal to one another. Although classical geometry does not include formal definitions for more complex shapes like horses or hands, it does provide procedures for establishing the geometric equivalence of different objects. For example, two polygons (or polyhedra) are said to be congruent if all of their corresponding edges are equal in length and all of the corresponding angles between edges are equal as well. Two polygons (or polyhedra) are said to be similar if all of the corresponding edges are in a fixed proportion with one another, and all of the corresponding angles between edges are equal.

**Figure 1.**
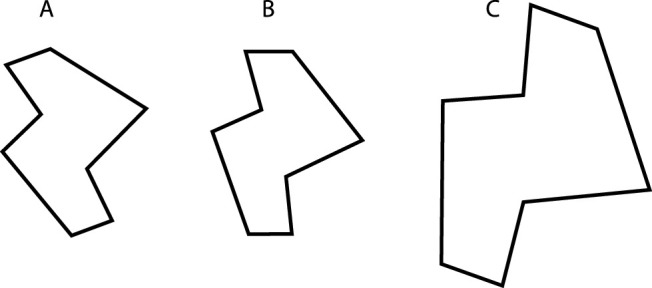
Three polygons that are geometrically similar to one another. Objects A and B are also congruent.

Figure 1 shows three polygons labeled A, B, and C. Whereas all three polygons are similar to one another, only A and B are congruent within classical Euclidean geometry. Most observers would agree, however, that A, B, and C all have the same shape. This suggests that the concept of similarity is closer to our intuitive notions of what constitutes shape equivalence than the concept of congruence. It is also interesting to note in this figure that all three polygons have different positions and orientations in space. Those variations are irrelevant to establishing the congruence or similarity between two polygons. It is only the relationship between the corresponding lengths and angles that matter. For the sake of simplicity, the examples in Figure 1 have been restricted to two-dimensional (2D) planar objects, but the same definitions are applicable to arbitrary numbers of dimensions.

Another more general way of defining geometric equivalence (i.e., congruence) in Euclidean geometry involves the possible alignment of one object with respect to another. Two objects are said to be congruent if and only if they can be aligned perfectly, so that each point on one object coincides with a corresponding point on the other and vice versa. Within Euclidean geometry, there are a restricted set of movements or transformations with which this alignment can be achieved. These are referred to as rigid transformations (or isometries), and they include translations, rotations, and reflections. What all these transformations have in common is that they do not alter the distances between any pair of points on an object. They only affect its overall position and orientation. It should also be pointed out, however, that reflection is slightly different from translation and rotation, in that it does not preserve handedness.

Expanding that set of transformations to include uniform dilations or scaling to achieve alignment provides a more general definition of the Euclidean concept of similarity. Uniform dilation alters the distances between all pairs of points on an object, but it does so in a fixed proportion.

### The *Erlangen program* of Felix Klein

Although Euclidean geometry was considered to be sacrosanct in philosophy and mathematics for over two millennia, that gradually started to change in the 19th century (Torretti, 2019). New geometries with assumptions different from Euclid's began to emerge, and older discoveries like projective geometry began to be recognized as alternatives to Euclidean geometry. In 1872, the German mathematician Felix Klein was awarded a professorship at the University of Erlangen at the young age of 23. In an effort to summarize his ambitious research goals, he wrote a booklet on the possible unification of all known geometries within a common framework, which is now referred to as the Erlangen program. His proposal relied heavily on group theory, which was not widely understood at that time. Klein argued that each type of geometry can be associated with a *group* of one-to-one transformations that map a space onto itself. The technical name for such a transformation is *automorphism* (e.g., Anderson & Feil, 2015). The geometric structures that are defined for each geometry are those that are invariant under its associated automorphism group. He also noted that these geometries can be organized in a hierarchical manner so that the group associated with one geometry can be a subset of the group associated with another. Klein's proposal was more like a manifesto than a formal proof, and the hard mathematical work to develop the theory with full rigor was left to others, especially to his friend and collaborator Sophus Lie.

We have already introduced how this works in Euclidean geometry. The Euclidean (isometry) group includes all combinations of translations, rotations, and reflections. If reflection is excluded from this set, the result is referred to as a special Euclidean group. Each individual transformation is an *isometry*—it preserves the lengths and angles on any geometric form. For example, to transform object A into object B in Figure 1, one must apply a translation and a rotation. It is easy to prove that two geometric figures are congruent (in the sense of having equal corresponding lengths and angles) if and only if there exists a transformation in the Euclidean group that makes the first figure identical to the second. If we are given an automorphism group, we can define congruence in terms of the equivalence classes that remain invariant with respect to arbitrary transformations in this group. In the case of the Euclidean group, all members of the same class have the same Euclidean shape and also the same absolute size. Additional invariants of Euclidean transformations include the perimeters and areas of 2D figures and the surface area and volumes of 3D objects.

If we expand the group, we create a more liberal congruence relation that partitions the space of geometric figures into coarser equivalence classes. This process is illustrated well by the transition from the Euclidean to the *similarity* group. The latter contains the Euclidean group as a subgroup but also includes all uniform dilations. All three objects in Figure 1 belong in the same equivalence class with respect to the similarity relation. Like metric congruence, similarity preserves all angles. Whereas absolute distances, areas, and volumes are not preserved over similarity transformations, the ratios of these attributes remain invariant, as do any translational, rotational, or reflective symmetries. Many mathematicians maintain that the similarity relation captures well the literal meaning of the everyday English expression that two geometric figures “have the same shape.” Thus, all circles have the same shape, all spheres have the same shape, all cubes have the same shape, and so on.

The preceding two paragraphs do not provide a mathematically rigorous definition of *shape* per se, but they do provide a widely used definition of *shape equivalence*. Two geometric figures *have the same shape* if and only if one of them can be transformed into the other via a similarity transformation—that is, via some combination of translation, rotation, reflection, and uniform scaling. The notion of shape that is defined by these equivalence classes is referred to as *Euclidean* (or, interchangeably, *metric*) shape throughout this article to distinguish it from alternative notions of shape.

**Figure 2.**
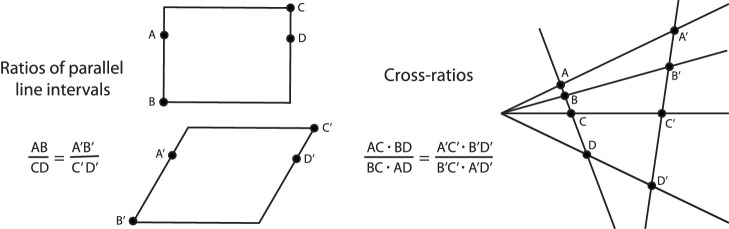
Some invariants of affine and projective geometry. The left panel depicts a rectangular object that has been subjected to a shearing transformation. Note that the ratios of parallel line intervals are preserved. The right panel shows the central projection of a line with four points labeled A, B, C, and D. The cross-ratio of line intervals defined by those points is invariant over all projective transformations.

To continue Klein's hierarchy, the next rung on the ladder is *affine geometry—*the study of the geometric properties that remain invariant under arbitrary *affine transformations*. The latter also form a group—the *affine group* of automorphisms. It contains the similarity group as a subgroup with the addition of nonuniform dilations, including shears. A shearing transformation is illustrated in the left panel of Figure 2. An appropriately chosen nonuniform dilation transforms a square into an arbitrary rectangle or rhombus. The equivalence relation defined by this group is more liberal than the similarity relation and partitions the space of geometric figures into coarser equivalence classes. For example, a circle is affine equivalent to an arbitrary ellipse. Thus, all ellipses (circles included) have the same *affine shape*. Also, all triangles have the same affine shape. Angle measures in general (including perpendicularity) are not meaningful concepts in affine geometry, but parallelism is an affine invariant. Another important invariant of the affine automorphism group is the ratio of lengths of parallel line segments (see Figure 2), including bisection. (The relative lengths of nonparallel segments are not preserved.) Concavities, convexities, and planarity are also invariant under affine transformations. Straight lines remain straight and all incidence relations are preserved.

The next rung on the ladder is *projective geometry*. It is generated by the group of *projective collineations*. A collineation is an automorphism that maps lines onto lines. If points A, B, and C are connected by a single line, then the projections of those points will also be connected by a single line. The set of all collineations forms a group that contains the affine group as a subgroup. In addition to collinearity and incidence, the so-called *cross-ratio* of four points is also a projective invariant (see Figure 2).

The final rung of the hierarchy is topology. This was not part of Klein's original proposal, but its inclusion is a natural extension of his ideas. Topology is the study of the geometric properties that remain invariant under continuous deformations such as stretching, twisting, crumpling, and bending. To illustrate, the outline of a human head is topologically equivalent to the outline of a hand, and a coffee mug is topologically equivalent to a doughnut. The invariants of topological transformations include incidence relations and the number of holes in an object.

Another more recent discovery is the concept of mixed groups. The simplest example of this is pictorial space. In the picture plane, distances and angles are well defined by 2D Euclidean geometry, but the depth dimension is different. All other planes in pictorial space (except the picture plane) are best characterized by a 2D affine geometry (Koenderink & van Doorn, 2012; Koenderink, van Doorn, & Wagemans, 2018), in which distances in different directions cannot be compared. Pictorial space is a mixture of these, and that is what causes the eyes or a pointed finger in a depicted scene to appear as if they are tracking the observer as he or she moves relative to the picture plane (Koenderink, van Doorn, Kappers, & Todd, 2001).

A summary of these different groups and a subset of their corresponding invariants is provided in Table 1. The most important thing to note in this table is its hierarchical organization. The topological invariants are also preserved by the projective group. The projective invariants are also preserved by the affine group. The affine invariants are also preserved by the similarity group, and the isometry group preserves the invariants of any of the other groups.

**Table 1.** Different transformation groups and some properties they leave invariant.

Object property	Isometry group	Similarity group	Affine group	Projective group	Topological group
**position**	variable	variable	variable	variable	variable
**orientation**	variable	variable	variable	variable	variable
**lengths**	**invariant**	variable	variable	variable	variable
**length ratios**	**invariant**	**invariant**	variable	variable	variable
**angles**	**invariant**	**invariant**	variable	variable	variable
**parallelism**	**invariant**	**invariant**	**invariant**	variable	variable
**parallel length ratios**	**invariant**	**invariant**	**invariant**	variable	variable
**colinearity**	**invariant**	**invariant**	**invariant**	**invariant**	variable
**cross-ratio** **s**	**invariant**	**invariant**	**invariant**	**invariant**	variable
**coincidence**	**invariant**	**invariant**	**invariant**	**invariant**	**invariant**
**closure**	**invariant**	**invariant**	**invariant**	**invariant**	**invariant**
**number of holes**	**invariant**	**invariant**	**invariant**	**invariant**	**invariant**

**Figure 3.**
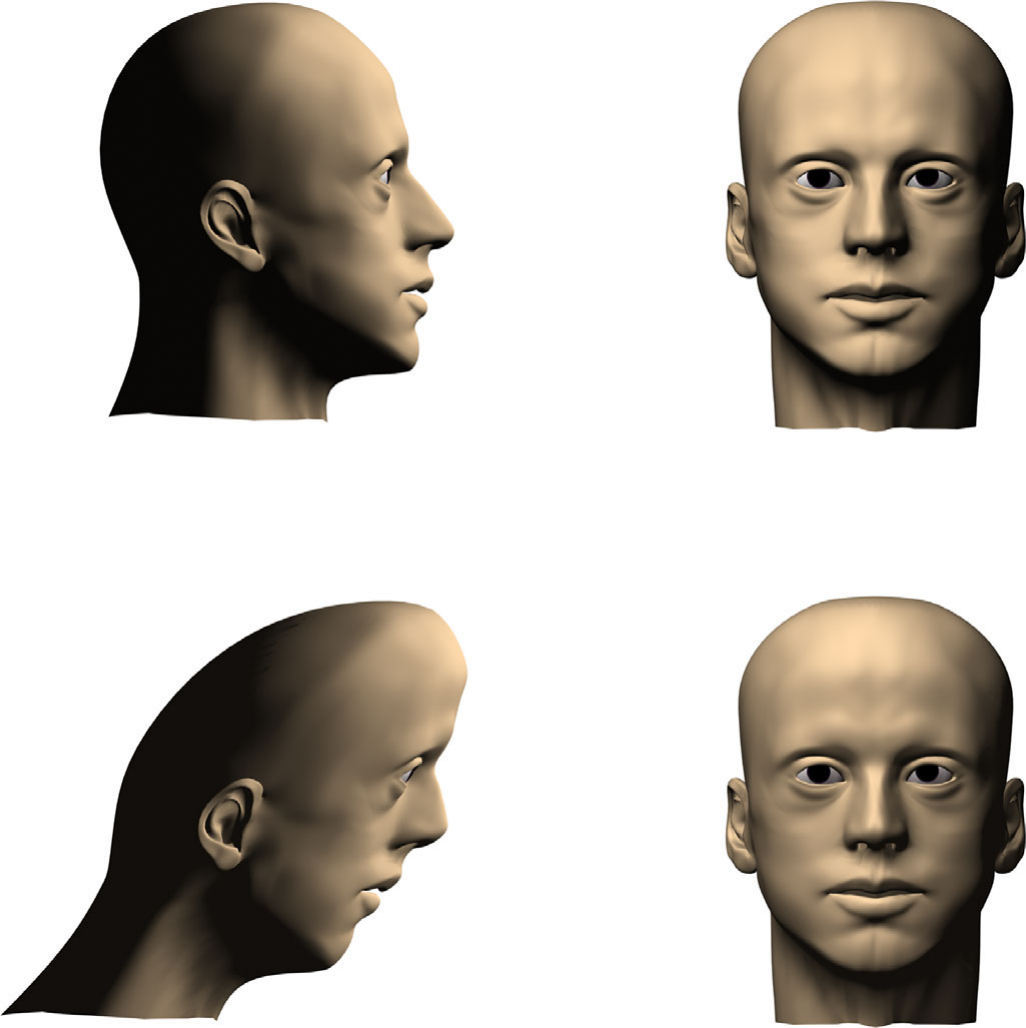
Front and side views of a normal human head (top) and one that was distorted by an affine shearing transformation. Note that the frontal views are indistinguishable from one another. This is an example of the bas-relief ambiguity first identified by Belhumeur, Kriegman, and Yuille (1997).

At this point, it may be reasonable to question what any of this has to do with the definition of shape, especially if we expect that term to be grounded in visual perception. After all, few if any observers would describe a circle and an ellipse as having the same shape, and they would likely report that a square and a trapezoid, or a head and a hand, are not even remotely similar in shape. However, these observations may be quite misleading because they involve transformations in the frontoparallel plane. There have been numerous mathematical analyses to show that visual information about 3D structure is often mathematically ambiguous. For example, early analyses by Koenderink and van Doorn (1991) and Todd and Bressan (1990) showed that two-frame apparent motion sequences of objects rotating in depth allow an infinite family of possible interpretations that are all related by an affine stretching or shearing transformation along the line of sight. A similar ambiguity was also demonstrated by Belhumeur, Kriegman, and Yuille (1997) for the analysis of 3D shape from shading. They referred to this as the bas-relief ambiguity because it nicely explains why bas-relief sculptures can appear to look perfectly normal when viewed from an appropriate vantage point. An example of this is shown in Figure 3, which shows a frontal and side view of a normal human head and one that has been subjected to an affine shearing transformation. Note that the shearing transformation is easily recognizable in the side view, but when the two heads are viewed from the front, they are perceptually indistinguishable. These theoretical analyses suggest that the perception of 3D shape from shading or motion is invariant within a subset of the affine transformation group for which unidirectional dilations or shearing transformations are oriented parallel to the observer's line of sight.

It is also interesting to note in this regard that the Klein hierarchy of geometries has a close connection to the concept of nonaccidental properties, which plays a prominent role in the literature on human perception and object recognition. Although that term was originally coined by Witkin and Tenenbaum (1983), it is now generally associated with Irv Biederman (1987) because it is a cornerstone of his famous theory of object recognition by components. One of the most fundamental problems in the perception of 3D shape is that the structure of a 2D image can be severely distorted by optical projection. However, if two objects can be distinguished by properties that are invariant over projection, then those properties might be especially informative for human or machine vision.

**Figure 4.**
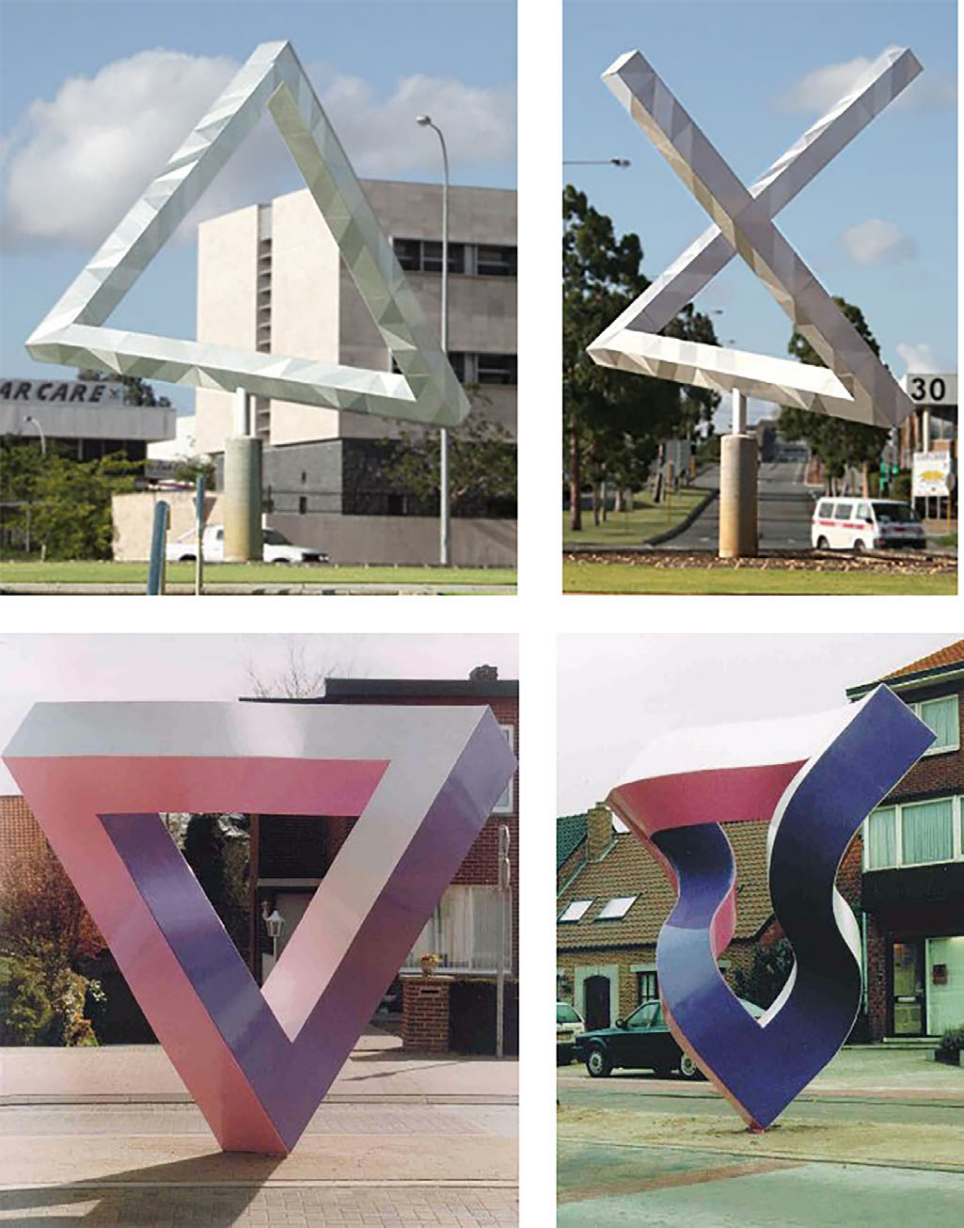
Impossible triangles created in two different ways. The top row shows a generic and accidental view of a sculpture by Brian McKay and Ahmad Abas (https://www.flickr.com/photos/themachobox/1068978352). The accidental view appears to have a cotermination of two bars that are actually separated in depth. The bottom row shows a generic and accidental view of a similar sculpture by Mathieu Hamaekers (https://im-possible.info/english/art/sculpture/hemaekers_unity.html). In that one, the accidental view appears to have straight edges, but they are actually curved in depth.

One such property includes coincidence relations (what Biederman refers to as cotermination), which are invariant over all topological transformations. It is possible that two noncoincident points may appear to be coincident in an image because they are aligned perfectly along the line of sight but that can only occur from a nongeneric view that is unlikely to occur by accident. There is considerable evidence that accidental views are perceptually interpreted as if they were generic, and that is the basis of many perceptual illusions (see top row of Figure 4). Another example of a nonaccidental property is collinearity, which is invariant over all projective transformations. Accidental views of curved contours that make them appear straight can also produce perceptual illusions (see bottom row of Figure 4). A third example discussed by Biederman is parallelism. There is a potential problem with parallelism as a nonaccidental property because it is only invariant under orthographic projection. Nevertheless, there is at least one famous illusion (i.e., the Ames room) that is caused by an accidental view of converging contours that makes them appear parallel.

The Klein hierarchy of geometries opens the door to a much broader set of equivalence relations for defining the concept of shape than could ever have been imagined within the narrow confines of Euclidean geometry. As we shall show in the discussion that follows, this expanded set of possible relations will be extremely useful for the analysis of shape in human perception. Indeed, there is abundant evidence to demonstrate that projective and topological relations are much more perceptually salient than Euclidean metric relations.

### Correspondence matching

**Figure 5.**
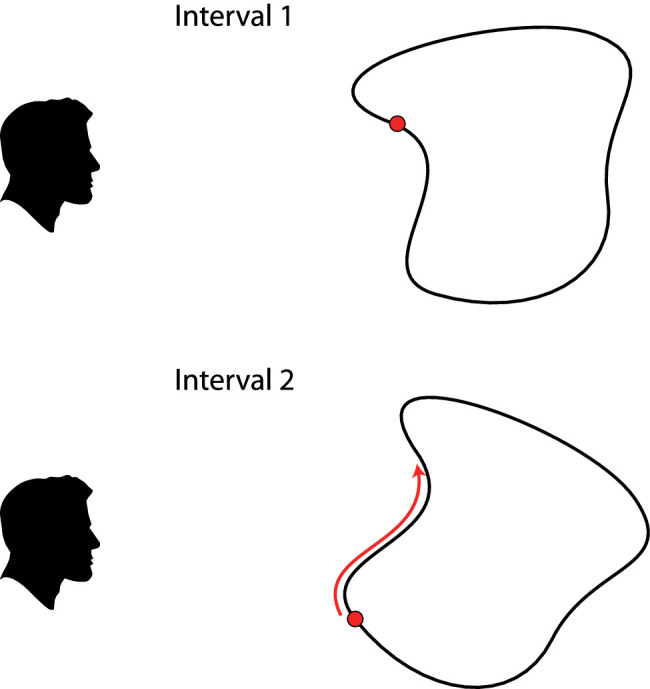
The correspondence matching task developed by Phillips, Todd, Koenderink, and Kappers (1997). Observers adjust the position of a dot on a surface so that it matches the position of a dot shown in an earlier interval.

It is interesting to note that almost all discussions of geometric equivalence between two objects involve a comparison of corresponding features, such as corresponding lines, corresponding angles, or corresponding points. This raises an interesting question about whether similar correspondence relations exist in human perception. The first experiment to investigate this issue was performed by Phillips, Todd, Koenderink, and Kappers (1997). At the beginning of each trial, a stereoscopic surface was presented with a single target location marked by a small dot, as shown in the upper panel of Figure 5. After a short interval, the same surface was presented at a different orientation, and the dot was moved to a different location, as shown in the lower panel of Figure 5. This second dot could be moved over the surface with a handheld mouse, and observers were required to move it to the same surface location as the target in the first view. They were able to perform this task with a surprising degree of precision. Under optimal conditions, the variance in their settings over multiple trials was just a few minutes of arc (see also Koenderink, Kappers, Pollack, & Kowato, 1997; Koenderink, van Doorn, Kappers, & Todd, 1997).

In describing their subjective impressions while performing this task, observers reported that there are certain salient structures on a surface, such as hills, valleys, and ridges, that can be used as landmarks for localizing the position of the probe dot on each trial. There are two important criteria that need to be satisfied for any surface point to be perceptually useful as a landmark. First, it must have some property that makes it stand out from its neighbors, and second, that property must be viewpoint invariant. Some possible candidates for landmarks include local maxima and minima in depth or orientation. Those points clearly stand out from their neighbors, but they change systematically when a surface is rotated in depth. Maxima and minima in curvature are much better candidates to serve as landmarks because they do not change as a function of the direction of view (see also Attneave, 1954).

In order to test that idea, Phillips, Todd, Koenderink, and Kappers (2003) presented surfaces at different orientations in depth and asked observers to mark the tops of the ridges and the bottoms of the valleys. Observers expressed great confidence in their abilities to perform this task, and the overall pattern of their responses had a high degree of reliability. Most important, the landmarks they selected were minimally affected by changes in surface orientation, thus indicating that their judgments were based on some property of the surface that was viewpoint invariant. The results also revealed that the marked locations had a negligible correlation with local extrema of depth or orientation and that they were highly correlated with local extrema of curvature.

More recent studies of correspondence matching have focused on 2D shapes (Norman, Phillips, & Ross, 2001; Schmidt, Spröte, & Fleming, 2016), and they have also expanded the original paradigm by including shape pairs that differ due to nonrigid deformations (Schmidt & Fleming, 2016). The results reveal that observers can reliably determine the point-to-point correspondences despite those deformations and that their responses can be accurately predicted by interpolating between perceptually salient landmarks based on extrema of curvature. When considered in combination, these findings provide strong evidence that observers are able to perceive the point-to-point correspondence between pairs of objects over a surprisingly broad range of conditions.

The primary take-home message from this research is that 2D and 3D objects contain sets of landmarks (sometimes referred to as singularities) that are defined by extrema of curvature and are invariant over changes in position and orientation (see also Atteneave, 1954; Hoffman & Richards, 1984). When considered in isolation, these local landmarks provide relatively little information, However, as we will describe more fully in later sections, the relationships between them provides a global topological scaffolding that makes it possible to decompose objects into perceptually distinct parts and to identify the point-to-point correspondence relations across different objects. Indeed, one of the most important distinctions among different representations of shape is the particular set of features they exploit and the manner in which their spatial arrangements are characterized.

### The measurement of shape differences

**Figure 6.**
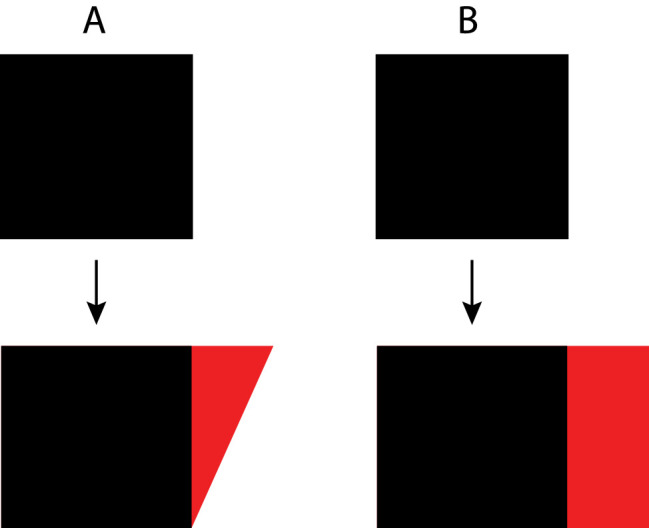
Two possible deformations of a square shape labeled A and B. The upper panel of each pair shows the original square, and the lower panel shows the transformed version of it. Note that some parts of the figures change while others do not. The changed regions are colored red.

**Objective measures of shape change** **.** There are many practical applications where it is important to quantify differences in shape, and there are many possible procedures by which this can be achieved. Because it is universally recognized that changes in the position, orientation, or size of an object have no effect on its objective shape, an ideal measure of shape change should not be affected by any of those transformations. Consider the two pairs of objects labeled A and B that are shown in Figure 6. The lower figure of pair A was created by displacing one vertex in a horizontal direction, and the lower figure of pair B was created by displacing an entire edge. In both cases, the original area of the figure is colored black and the changed region is colored red.

One possible metric for evaluating shape differences between objects is called the overlap index. It defines shape change on a scale of 0 to 1 based on the ratio of areas (or volumes) between the intersection of two objects and their union (Lee & Sallee, 1970; Zhao & Stough, 2005). For the examples shown in lower panels of Figure 6, the area shown in black is the intersection, and the combined areas of the black and red regions are the union. Note that the proportion of overlap is larger for the pair of objects on the left (labeled A), so they have a smaller shape difference according to this measure than the pair of objects on the right (labeled B). An important limitation of the overlap index is that the objects to be compared must be aligned and scaled appropriately so that the area of overlap is maximized. Lee and Sallee (1970) argued that for all possible translations, rotations, and size changes, there exists one that maximizes the ratio of overlap for the two objects, and that is the one for which the overlap index should be computed. However, they did not provide a specific method for determining that special alignment. This is sometimes referred to as the Procrustes problem, after the mythical Greek bandit who made his victims fit his bed either by stretching their limbs or cutting them off.

Efficient algorithms for solving the Procrustes problem have been developed more recently (e.g., Dryden & Mardia 2016; Hamsici & Martinez, 2008; Kendall, 1984). These algorithms are designed specifically to compare the shapes of objects that have corresponding sets of landmarks. Each configuration of landmarks can be defined as a point on a hypersphere, whose dimensionality is determined by the number of landmarks. A least squares procedure is employed to find the optimal translation, rotation, and scaling of one configuration relative to the other to minimize the geodesic distance between them on the hypersphere. The shape difference between two objects is defined by the residual geodesic distance following their optimal alignment. According to that measure, the shape difference between the pair of objects depicted in Figure 6A is smaller than the pair of objects depicted in Figure 6B.

Another possible measure for scaling differences between shapes involves comparing the angles at each corresponding vertex on polygons or the angles across each corresponding edge for polyhedra. This can be achieved in a variety of ways, such as computing the sum of squares for the difference between each corresponding angle. Note that this approach does not require any special alignment between the objects to be compared, but it does require a correspondence mapping between the vertices (or edges) on each object. It is also limited to polygons or polyhedra with the same number of vertices. For the two shape changes depicted in Figure 6, an analysis of the corresponding angles would show that the shape difference shown in Figure 6A is much larger than the one in Figure 6B. Indeed, according to that measure, the two objects in Figure 6B have exactly the same shape. Other possible shape difference metrics include measures of convexity (e.g., the perimeter of an object divided by the perimeter of its convex hull) or a comparison of object topology using the Euler characteristic. Neither of those measures would detect any shape change at all among the objects depicted in Figure 6.

There are far too many shape difference metrics used in the literature to provide an exhaustive account here, but an excellent summary of many of them has been provided by Wirth (2004). We have chosen these particular examples to demonstrate that there is a surprising degree of inconsistency among existing shape difference metrics. With respect to the shape changes shown in Figure 6, some of them would reveal that the shape change depicted in Figure 6A is larger than the one shown in Figure 6B. Others would reveal exactly the opposite or fail to detect either of the depicted shape changes in that figure. Some measures require a prealignment of objects or a correspondence map in order to be effective, whereas others do not. The problem is that all of these metrics have been designed to measure particular aspects of shape in specific contexts, but there is no overarching analysis to bind them together in a coherent manner. One possible method to achieve that goal might be to scale shape changes in terms of their perceptual discriminability.

**Perceptual measures of shape change** **.** In a remarkable study published almost 40 years ago, Chen (1982) proposed a radical new hypothesis that the relative perceptual salience of different types of shape change may be systematically related to the Klein hierarchy of geometries. He referred to this as the topological approach to visual perception, based on his observations that changes to topological properties are easier to detect than any other type of shape change (see also Chen, 1982, 1983; He et al., 2015). Chen has provided numerous psychophysical examples to support this hypothesis, but he has never employed objective measures of shape change to see if any of them could potentially account for his results.

**Figure 7.**
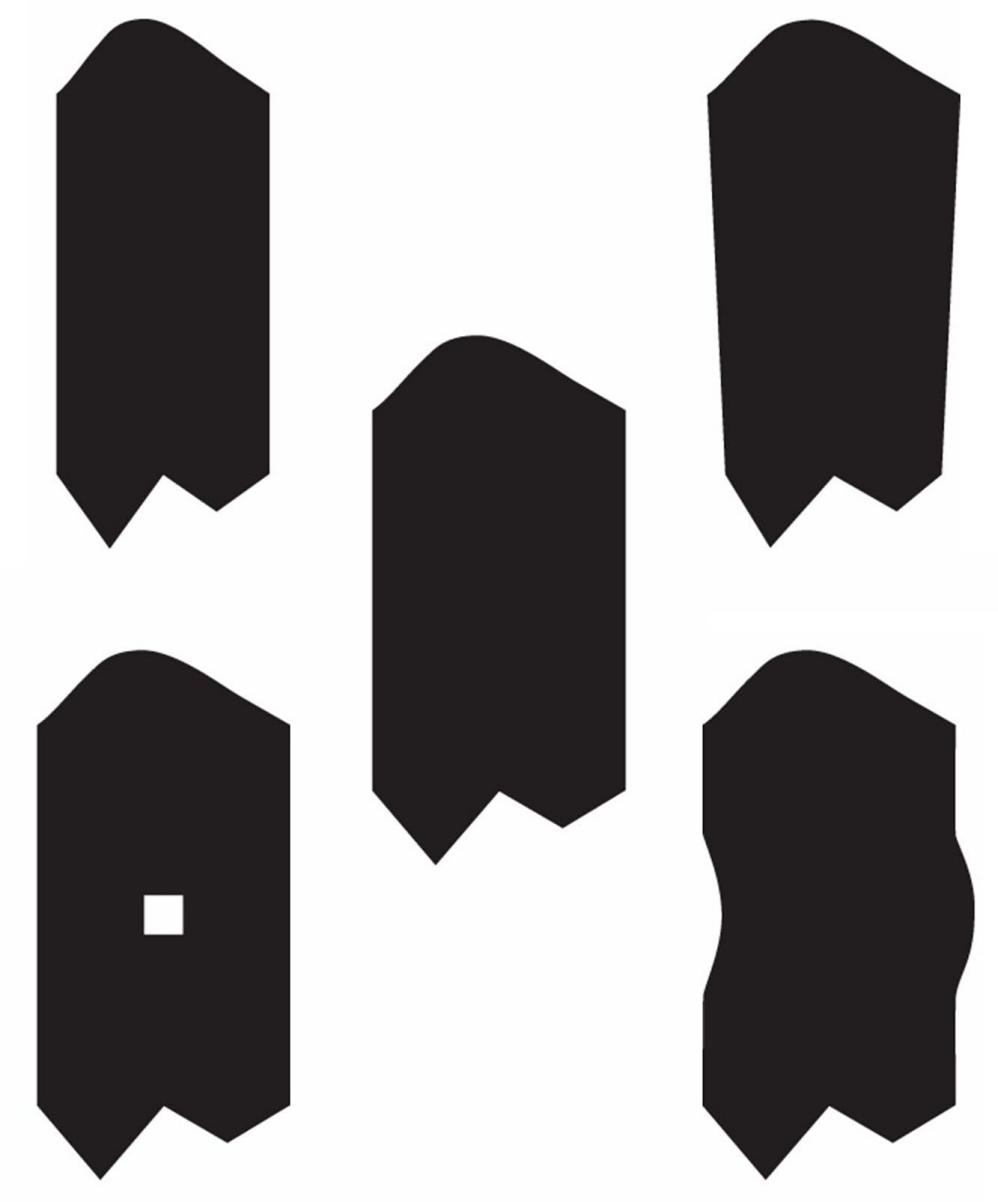
Some example stimuli used to study the perceptual salience of different types of shape change. All of the shapes in the periphery involve distortions of the one in the center. Moving clockwise from the upper left, the depicted distortions involve a change in the aspect ratio, making parallel lines converge, adding curvature to straight line segments, or punching a hole in an object. (Reprinted from Todd, Weismantel, & Kallie, 2014.)

A recent experiment by Todd, Weismantel, and Kallie (2014) was designed to address this issue. Observers were asked to discriminate 2D forms that could be subjected to four different types of shape change as shown in Figure 7. These included punching a hole in an object (a topological property), adding curvature to a straight edge (a projective property), changing the relative orientations of parallel edges (an affine property), and changing the overall aspect ratio of an object (a Euclidean metric property). Each type of shape change could be presented with varying magnitudes, and the method of constant stimuli was used to determine the 75% threshold for detecting each one. In addition, nine possible shape difference metrics were employed to evaluate the objective magnitudes of shape change at threshold for each of the possible transformations.

The psychophysical results provided a dramatic confirmation of Chen's hypothesis. No matter what metric was employed to measure the thresholds on a common scale, the thresholds for the Euclidean metric changes were 10 to 30 times larger than those obtained for the topological changes. The thresholds obtained for the affine and projective changes were both in between those, but the projective changes produced consistently smaller thresholds than the affine ones.

An earlier experiment by Biederman and Bar (1999) produced similar results for images of 3D objects. Unlike Todd, Weismantel, and Kallie (2014), they did not employ any objective measures of shape change. The calibration method they used was to select different pairs of objects with different types of shape change that were all equally discriminable when viewed together at the same orientation in depth. However, when one of the objects was rotated in depth relative to the other, the changes to nonaccidental properties were much easier to detect than the Euclidean metric changes. In their analysis of the results, Biederman and Bar combined all the affine, projective, and topological changes into a single category, so it is not possible to determine from their reported data whether there were any systemic differences within that group.

Another related experiment using 3D stereoscopic objects was performed by Todd, Chen, and Norman (1998). The stimuli depicted wireframe figures in varying orientations consisting of four line segments. Observers were required to discriminate pairs of objects using a match to sample task. The foil in each case was created by rotating one line segment by exactly 40 degrees relative to the others. In one third of the trials, the rotated line segment altered the pattern of intersection in 3D space (a topological property). In another third, it altered whether or not three of the line segments were coplanar (an affine property), and in the remaining third, it altered the Euclidean metric structure of an object while leaving its affine and topological structure intact. The percentage of correct responses was 80% in the Euclidean condition, 90% in the affine condition, and nearly 100% in the topological condition. Similarly, the average reaction time was 3 seconds in the Euclidean condition, 2 seconds in the affine condition, and only 1 second in the topological condition.

When considered in combination with Chen's (1982, 1985, 2005) many experiments on pop-out, these results provide compelling evidence to support his hypothesis about the relative perceptual salience of different types of transformations. Changes in shape that alter the topological structure of an object are easier to detect than changes that alter its projective structure while leaving its topology intact. Changes that alter projective structure are easier to detect than changes in affine structure that leave projective relations intact. The most difficult changes to detect are those that only affect Euclidean metric structure. This is why the type of change depicted in Figure 6A is easier to detect than the one depicted in Figure 6B. The change in Figure 6A alters the affine property of parallelism, whereas the one in Figure 6B does not. We know of no objective measures of shape change that are able to predict this overall pattern of sensitivity. Indeed, almost all of them predict exactly the opposite.

### Identifying different types of shape change

**Figure 8.**
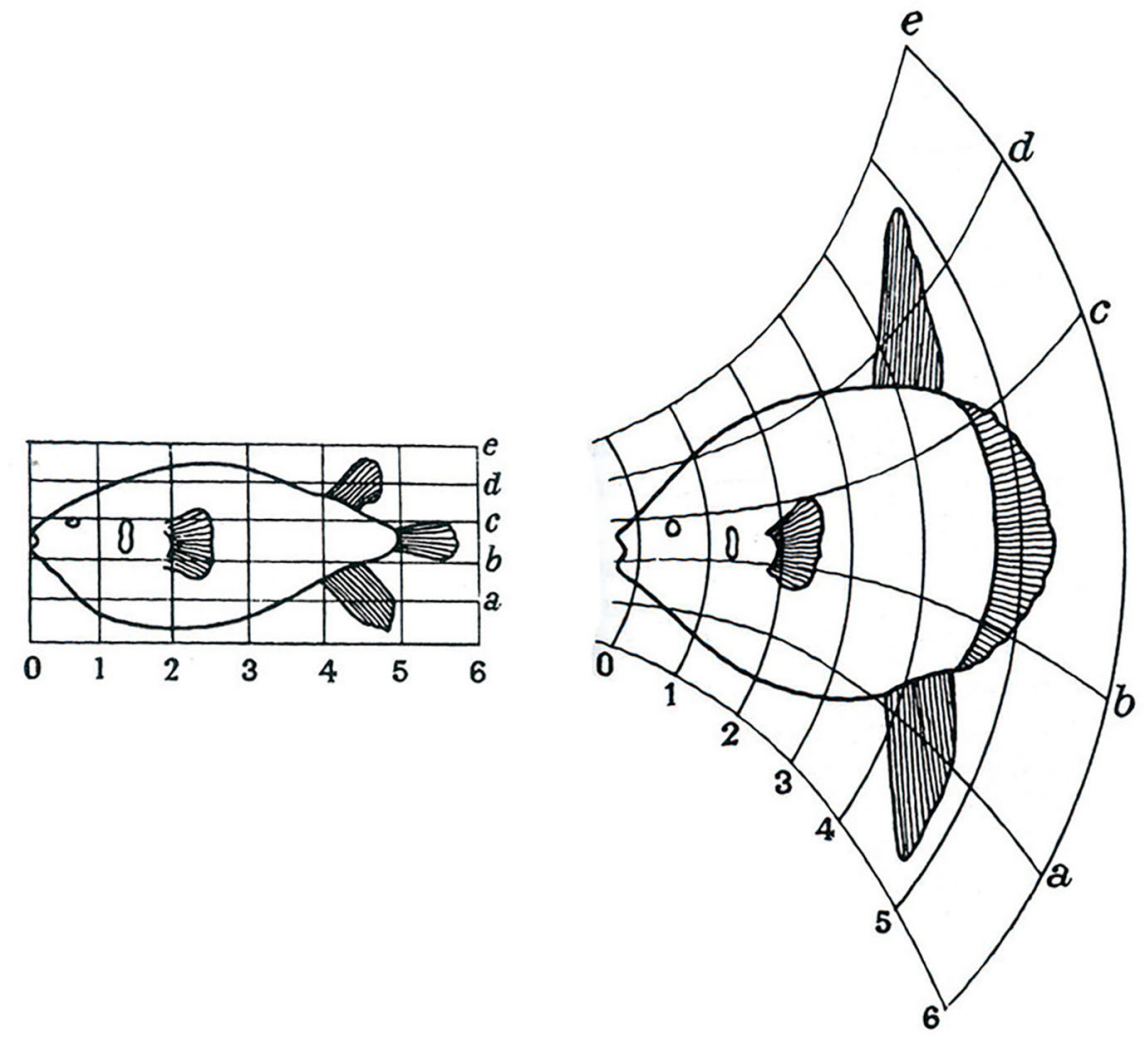
The evolutionary shape change between diodon on the left and orthagoriscus on the right. (Reprinted from *On* *Growth* *and* *Form* by D'Arcy Wentworth Thompson, 1917.)

It is often insufficient to know that two shapes are different without also knowing how they are different. There is a considerable body of research on the classification of shape changes in many different fields, including biology, engineering, and human perception. Let us first consider the shape changes that occur in complex biological forms due to growth or evolution. The first systematic analysis of these changes was performed by the great Scottish naturalist D'Arcy Wentworth Thompson (1917) in his famous book *On* *Growth* *and* *Form*. The method he employed for comparing 2D shapes involved covering them with a rectangular grid to provide a coarse coordinate system. He would then distort the grid on one so that the most salient landmarks on both shapes were located at corresponding positions on their respective grids (see Figure 8). The pattern of grid distortion required to make that happen highlighted the underlying geometric transformation by which the two forms were related.

**Figure 9.**
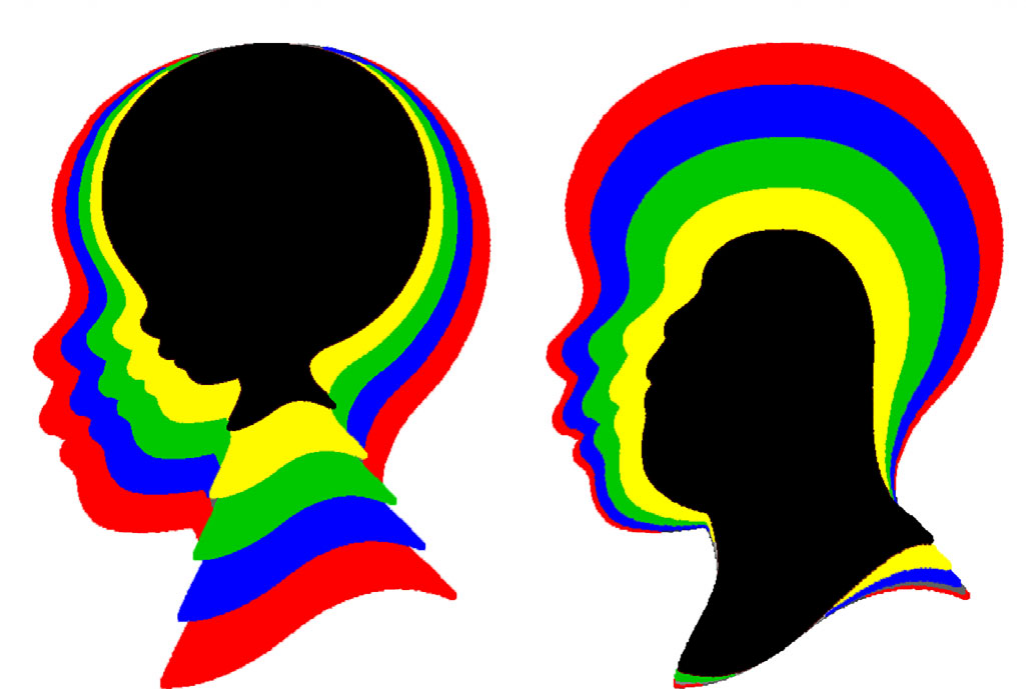
Two sequences of human head profiles. The one on the left was generated using a cardiodal strain transformation to simulate normal human growth. The one on the right was created with a modified version of that transformation that simulates human evolution.

The significance of Thompson's insights for human perception was first recognized in an influential study by Shaw, McIntyre, and Mace (1974). They were intrigued by Thompson's discussion about the growth of a human head, and they developed a mathematical transformation called cardiodal strain that allowed them to simulate how the shape of a head changes as a function of age. This was later modified slightly by Todd and Mark (1981) to better capture the size changes that also occur due to human growth (see Figure 9), and it allowed them to accurately fit longitudinal x-rays of human heads at different ages. The underlying physical basis of this transformation is that bone grows proportionally to the pressure that is placed upon it (Wolff, 1892/1986)—what is often referred to as Wolff's law.

When arrays of transformed head profiles are presented to human observers, the ones transformed by cardiodal strain are readily identified as growth, whereas those depicting other control transformations are not (Mark & Todd, 1985; Mark, Todd, & Shaw, 1981; Pittenger & Shaw, 1975; Todd, Mark, Shaw, & Pittenger, 1980). Interestingly, the same results are obtained if observers are presented with sequences depicting dogs, monkeys, or Volkswagen Beetles (Pittenger, Shaw, & Mark, 1979), thus indicating that cardiodal strain is a general style of change that can be perceptually identified over a wide variety of objects. It can also be generalized to 3D objects or shaded photographs of faces (Mark & Todd, 1983).

**Figure 10.**
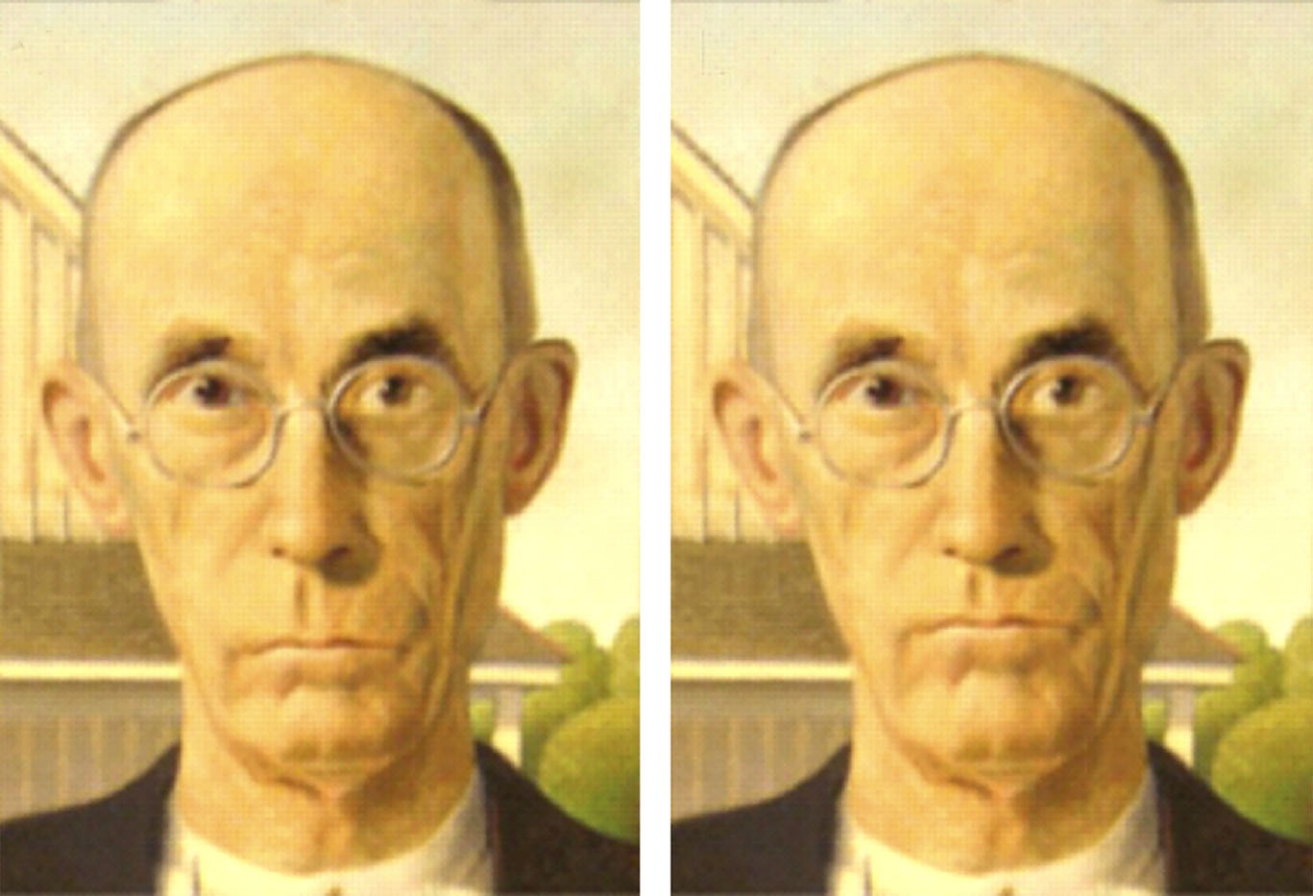
The left panel shows a man's face from the painting *American* *Gothic* by Grant Wood (1930). The right panel shows a transformed version in which the distance between the eyebrows and the mouth was reduced. The original image on the left is judged to have a sad expression, whereas the one on the right is judged as angry. (Reprinted from Neth & Martinez, 2009.)

Research on the perceptual categorization of shape change fell out of favor in the 1990s, but it has been making a comeback in recent years. One particularly exciting application of this approach involves the perception of emotional expression (Neth & Martinez, 2009; Martinez, 2017). Their analysis begins with the automatic extraction of facial landmarks such as the eyes and mouth, and the distances between all pairs of landmarks are measured. These distances can then be systematically distorted by a set of possible geometric transformations. Each basic facial expression is associated with a specific transformation relative to a neutral face, and these can be applied in combination to create compound expressions such as happily surprised, which are easily recognizable (Du, Tao, & Martinez, 2014). Figure 10 shows an example from Neth and Martinez (2009). The left panel depicts a face from the painting *American Gothic* that is perceived to have a sad expression even though there is no downward curvature of the mouth. The right panel shows a transformed version of this face, in which the vertical distance between the brows and the mouth has been reduced. This causes the transformed face to appear angry.

Similar experiments have also been performed with nonbiological transformations. An especially interesting variation of this research has been reported by Schmidt and Fleming (2018). They presented observers with a photograph of a single object and asked them to identify a physical process that could have affected its shape, such as being folded, crumpled, twisted, or bent. Both free response and discrimination procedures were employed. The results revealed that observers can make these judgments with a surprising degree of reliability. This suggests that there are particular features of an object's shape that provide information about how it may have been altered in the past (see also Fleming & Schmidt, 2019; Leyton, 1992, 2012; Schmidt, Phillips, & Fleming, 2019).

It is reasonable to question whether all of the transformations described above should actually be considered changes in shape. They certainly are by the classical definition from Euclidean geometry that a shape change is anything that cannot be undone by translations, rotations, and dilations, but is that definition consistent with our intuitive understandings about shape? Consider the ability of normal observers to recognize the shape of a human head (see Figures 3, 9, and 10). We are able to identify head shape over a wide range of different individuals, with different facial expressions, hairstyles, or clothing accessories. We can also identify head shape across people of different ages, genders, or races or even when someone is depicted in a caricature or a cubist painting. These observations suggest that the perception of head shape must be based on some remarkably abstract property that is invariant over all the possible different forms that are recognizable as human heads. This is also true for categorizing other objects such as houses or airplanes, because they too can appear in a wide variety of different forms. If shape is the primary attribute for recognizing objects like houses or airplanes, as is widely believed, then the concept of shape must be flexible enough to accommodate all of their possible variations. The traditional view from classical Euclidean geometry is incapable of achieving that.

A closer examination of Figures 3, 9, and 10 reveals that all of the transformed versions of these objects have one thing in common. They all share the same set of parts. For example, the transformed heads in Figure 9 all have a chin, mouth, nose, and cranium, and the depicted transformations involve relatively subtle changes in the spatial arrangements of those parts. The concept of parts does not exist in classical geometry, but observers often refer to them in their verbal descriptions of shape. This suggests that parts may be an important component in the perceptual representation of shape.

## The representation and measurement of shape

All of the discussion provided thus far has focused on the evaluation of shape equivalence and the measurement of shape differences, but these issues shed little light on the definition of shape per se. In his influential book on *Theoretical* *Geography*, William Bunge (1962) proposed four criteria for evaluating any measure of shape: (1) It should be objective; (2) tt should not consist of something less than shape, such as a set of position coordinates; (3) it should not consist of something more than shape such as a set of parameters for a Fourier or Taylor series; and (4) it should not do violence to our intuitive notions of what constitutes shape. In order to satisfy this fourth criterion, any two objects that are similar according to a valid measure of shape should also be perceptually similar and vice versa.

We believe that Bunge's fourth criterion should be elaborated in more detail in order to highlight some specific aspects of human performance that have been reported in the literature. For example, we know that human observers can identify shape differences quite rapidly (see Biederman & Bar, 1999; Chen, 1982, 1985, 2005) and that some types of shape change are easier to detect than others (Todd, Chen, & Norman, 1998; Todd, Weismantel, & Kallie, 2014). There is also extensive evidence that human observers decompose objects into perceptually distinct parts (see Biederman, 1987; De Winter & Wagemans, 2006). A perceptually valid theory of shape representation should be able to explain all of those findings.

### Generic data structures

#### Maps

One common method for representing objects in the environment involves a type of data structure called a “local property map.” The basic idea is quite simple and powerful. A visual scene is broken up into a matrix of small local neighborhoods, each of which is characterized by a number (or a set of numbers) to represent some particular local property, such as position, orientation, or color. One major shortcoming of local property maps as a possible data structure for the perceptual representation of shape is that they are highly unstable. Consider what occurs, for example, when an object is translated or rotated in space. This causes all the positions and orientations within each local neighborhood to change, so that the representation does not exhibit shape constancy.

#### Graphs

Another type of data structure that can be used for the representation of shape is called a graph. A graph is a set of nodes called vertices and a set of connections between those nodes called edges. The history of graph theory is often traced to a article works published in 1736 by Leonard Euler on the “Seven Bridges of Königsberg.” Euler also developed a formula relating the number of edges, vertices, and faces of a convex polyhedron that is generally recognized as the beginning of topology. The appeal of graphs in vision science stems from the fact that connected patterns of visible contours in line drawings can provide considerable information about the 3D shapes of objects, even in the absence of any shading or texture. This suggests that a graph-based analysis could potentially provide a representation of shape that is closer to our perceptual intuitions than the set of position coordinates in a local property map.

### The representation of 2D shape

#### Contour segmentation at extrema of negative curvature

In an early article on shape perception, Atteneave (1954) argued that information about object shape in contour drawings is not distributed homogeneously in an image but is instead concentrated around curvature extrema. To demonstrate this, he modified an image of a sleeping cat in which curved contours were replaced by straight line segments that all terminated at the locations of curvature extrema from the original image. Despite these changes, this modified drawing is instantly recognized as a cat. This idea has more recently been developed by Feldman and Singh (2005), who showed that the Shannon information for curved image contours is actually much greater for negative extrema than for positive ones.

We have already discussed how curvature extrema provide salient landmarks on an object for establishing point-to-point correspondence relations, but they also provide information for segmenting contours into perceptually distinct parts. Hoffman and Richards (1984) were among the first to argue that decomposing shapes into parts is a basic function of human perception and that negative minima of curvature are ideal features to define part boundaries. This idea has been tested extensively over the past 35 years, and it has received considerable empirical support (see Singh, 2015, for a review). One study by De Winter and Wagemans (2006) is especially noteworthy in this regard because of its impressive scope. They asked 201 observers to segment the outline drawings of 88 common objects. The results revealed that locations of negative curvature were often selected as segmentation points but that observers’ judgments were also influenced by several other factors, such as the lengths of the segmentation boundaries.

It is also interesting to note in this context that the curvature singularities along the boundary of an object can be represented as a primitive form of graph based on their adjacency relationships. A given Feature A might be described, for example, as being clockwise to the right of Feature B and counterclockwise to the left of Feature D. Indeed, that is precisely the type of language that observers use when describing how they perform correspondence matching tasks.

#### Blum's medial axis

Another way of representing the overall shape and part structure of a 2D figure is the medial axis transform first proposed by Blum (1967, 1973). The medial axis of a figure can be conceptualized in several different ways, but probably the most intuitive of these is called the maximal disk model. A maximal disk is one that touches the boundary of a planar shape in at least two locations without crossing it. The medial axis is defined as the locus of center points for all possible maximal disks on a figure. An alternative conceptualization is called the grassfire model. Suppose that the boundary of a grass area is set on fire. The medial axis in that case is defined by the points where the fires from different portions of the boundary come together.

**Figure 11.**
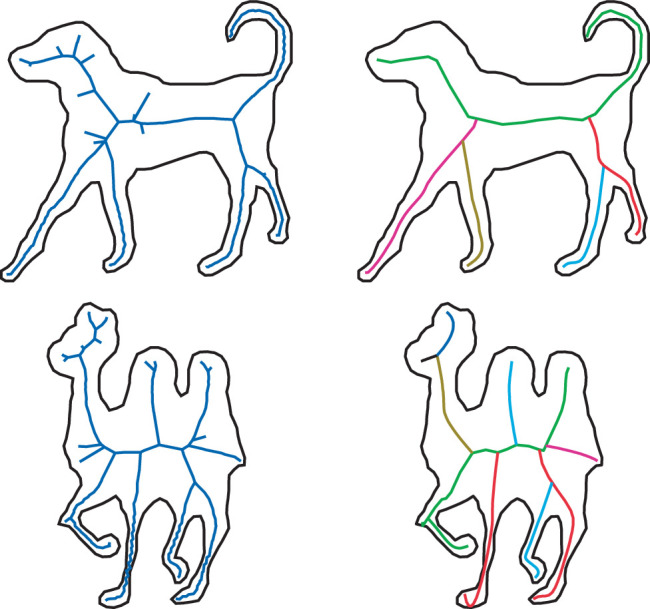
The medial axes of a dog and a camel computed using a traditional method (left) and a Bayesian estimation procedure. (Reprinted from Feldman and Singh, copyright (2006) National Academy of Sciences, U.S.A., with permission. This material is excluded from any creative common license.)

The medial axis transform has been studied extensively in computer vision (see Feldman & Singh, 2006, for a review), and the results have shown that it is notoriously unstable over small perturbations of the boundary. It can also produce branches that bear no resemblance to the part structures perceived by human observers (see left column of Figure 11). In an effort to correct these problems, there have been numerous attempts to prune unwanted branches from the output of the computation. Feldman and Singh (2006) have developed a particularly promising approach to achieve that goal using a Bayesian estimation procedure to compute medial axes (see right column of Figure 11).

The Blum medial axis transform provides a more complex graph structure than the adjacency relations between contour singularities. Note in Figure 11 that the branches created by this process have a hierarchical nested structure, which provides a natural framework for subdividing an object into parts. Although it would be possible to explicitly represent the relative lengths of these branches (e.g., to encode the difference between a gazelle and a giraffe), the medial axis is primarily a topological structure, and, as such, it is invariant over a wide range of possible object deformations.

#### Shock graphs

An interesting extension of the medial axis transform has been proposed by Kimia, Tannenbaum, and Zucker (1995) and Siddiqi, Shokoufandeh, Dickenson, and Zucker (1999). Their model uses a weighted combination of the grassfire equation of Blum with the heat (diffusion) equation of thermodynamics. These define a curve evolution process that gradually deforms the shape of an object. There are several types of singularities (called shocks) that can arise as a closed curve is deformed in this manner. First-order shocks are associated with protrusions or indentations along a contour. Second-order shocks consist of thin necks that connect two blobs on each side, which are intuitively recognized as parts, and third-order shocks produce a bending of extended regions.

The inward evolution process described above creates a topological skeleton of a 2D shape, much like the grassfire equation of Blum. However, it provides two important enhancements over more traditional methods (Siddiqi, Shokoufandeh, Dickenson, & Zucker, 1999). First, the individual nodes of the shock graph are labeled by the types of singularity from which they arise, and second, they are also labeled with respect to the time of their appearance within the overall process of curve evolution. This provides a much richer data structure that can be used for matching shapes or subdividing them into parts. There is also some psychophysical evidence that shock-based descriptions are predictive of human shape perception. Siddiqi, Kimia, Tannenbaum, and Zucker (2001) have shown that observers’ shape judgments on a variety of tasks can be influenced by when shocks form with respect to one another and also by where on the boundary they form.

#### Feature vectors

There is another type of 2D representation that is used frequently in neural network models of object recognition (Fukushima, 1980). This approach is loosely based on the hierarchical structure of the primate visual system. Visual inputs are initially convolved with a bank of linear filters, and the output of that process then undergoes a sequence of additional convolutions and normalization processes. In the initial stages of this analysis, these filters will only respond to simple patterns within a localized region of visual space. However, at higher levels, they tend to respond to more complex patterns irrespective of their precise locations. These models are typically trained using supervised learning on a large set of input images. This causes the filters at higher levels of the network to become tuned to abstract features that best separate the training images into separate categories. One well-known example of this approach is the HMAX network by Riesenhuber and Poggio (1999) and Serre, Oliva, and Poggio (2007). The performance of convolutional neural networks (CNNs) has expanded greatly in recent years as it has become technologically possible to add more and more layers to these networks. Two of the more prominent recent architectures include AlexNet (Krizhevsky, Sutskever, & Hinton, 2012) and GoogLeNet (Szegedy et al., 2014).

**Figure 12.**
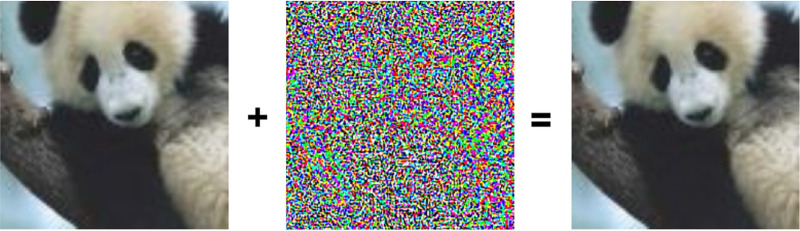
An example of an adversarial image. The left panel shows the image of a panda. The middle panel shows a pattern of noise that has been optimized so that it is categorized as a gibbon by GoogLeNet with the highest possible confidence. The right panel shows the image on the left with a small amount of noise from the middle panel added to it. This image is categorized as a panda by human observers and as a gibbon by GoogLeNet. (Reprinted from Goodfellow et al., 2017, available for use through open access.)

Although CNNs typically outperform other types of algorithms for object recognition in annotated data sets, they should not be considered plausible representations of shape per se. One important reason for this is that they are not exclusively sensitive to the shapes of objects. They can also base their categorization decisions on other image attributes such as color, texture, or the pattern of shading. It is also interesting to note that the images that maximize the classification probability for a given response category are often visually uninterpretable to human observers. This is the property that allows the creation of adversarial images that are categorized as one thing by human observers and something entirely different by a CNN. A good example of this from Goodfellow et al. (2017) is shown in Figure 12. The left panel shows the image of a panda. The middle image was created by using iterative optimization algorithms to propagate information backward through the network and synthesize an image that maximizes the probability to be classified as a gibbon. Note that it appears perceptually as random noise. If a small amount of this noise is added to the image of the panda as shown in the right panel, it has no effect on observers’ judgments but tricks the network into categorizing the combined image as a gibbon with a high level of confidence.

**Figure 13.**
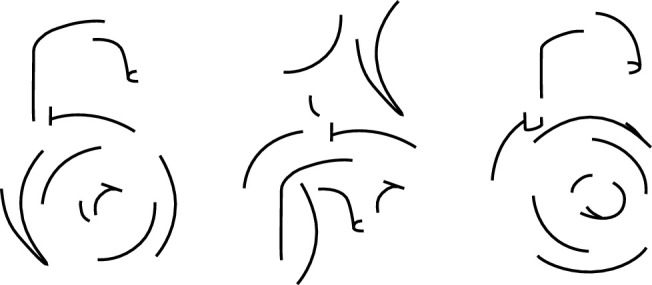
Three images from a match to sample task. The sample in the left panel contains half the contours from a drawing of a lock. The middle panel shows a spatially scrambled version of the sample, and the right panel shows a complementary version that contains all of its deleted contours. Human observers choose the complementary version as most similar to the standard, whereas the HMAX network chooses the spatially scrambled version. (Re-created from Hayworth, Yue, & Biederman, 2007, with permission from the authors.)

Another potential problem with CNNs for the representation of shape is that they seem to violate some of the central principles of Gestalt psychology. For example, human observers can spontaneously detect global shapes based on the alignments of local oriented features that are spatially separated from one another (e.g., Biederman, 1987; Field, Hayes, & Hess, 1993; Grossberg & Mingolla, 1985; Grossberg, Mingolla, & Ross, 1997; Kellman, Garrigan, & Shipley, 2005; Petrov, Van Horn, & Todd, 2011). The importance of feature arrangements for human observers relative to neural networks was nicely demonstrated in a classic experiment by Hayworth, Yue, and Biederman (2007). They created line drawings of common objects and broke them up into complementary pairs, each of which contained half the contours from the original (see Figure 13). They then performed a match-to sample task with human observers and the HMAX network. The sample image was always one of the complementary drawings. The comparison images always included a scrambled version of the sample together with the complement of the sample. Observers always judged that the complement appeared more similar to the standard, whereas HMAX always selected the scrambled version as being more similar. This finding suggests that the network treats the images as a set of features without considering how they are organized.

Human observers are quite good at recognizing objects that are partially occluded, and they can also recognize objects in different poses or against different backgrounds. However, these changes in viewing context often produce miscategorizations by CNNs (Alcorn et al., 2019; Kortylewski et al., 2020; Yuille & Liu, 2021; Wang et al., 2018; Zhu et al., 2019). All of these findings provide strong evidence that the performance of current CNNs may be fundamentally different from human perception. Kortylewski et al. (2020) have recently argued that it may be possible to achieve greater tolerance to changing contextual conditions by training a network to detect parts of objects, rather than representing each category with a single feature vector. We suspect this approach may produce results that are much closer to human perception, but it would also be necessary to encode how those parts are arranged with respect to one another.

Although feature vectors are a powerful tool in computer vision for object recognition, they do not satisfy Bunge's second and fourth criteria for a good measure of shape. The features that are extracted by these models are not a pure measure of shape because they also encode the location, orientation, size, and color of an object. Similarly, these models cannot explain why some types of shape change are more difficult to detect than others, nor do they provide an easy method of subdividing an object into perceptually distinct parts.

## The representation of 3D shape

### Depth and orientation maps

The first explicit representation of 3D shape within vision science was proposed over 70 years ago by James Gibson (1950). He argued that our immediate knowledge of visible surfaces can be described as a point-by-point mapping of depth and orientation for each local surface region within the field of view. Many years later, this same type of representation was adopted by researchers in machine vision. For example, in his pioneering work on shape from shading, Horn (1975, 1977) defined shape as a local orientation map (see also Lee & Rosenfeld, 1985; Pentland, 1984; Witkin, 1981). A similar approach was also adopted by Marr (1982) and Marr and Nishihara (1978). They argued that our immediate perceptual awareness of surfaces is based on local mappings of depth and orientation, and they named this representation the 2 1/2D sketch.

These theoretical ideas about the structure of visual knowledge have important methodological implications. In any psychophysical experiment on 3D form perception, an observer must be asked some question about the perceived structure of the stimulus. If it is assumed, for example, that our immediate awareness of surfaces is best described as a local depth or orientation map, then the most sensible psychophysical procedure for studying the perception of surfaces would involve judgments of local depth or orientation.

**Figure 14.**
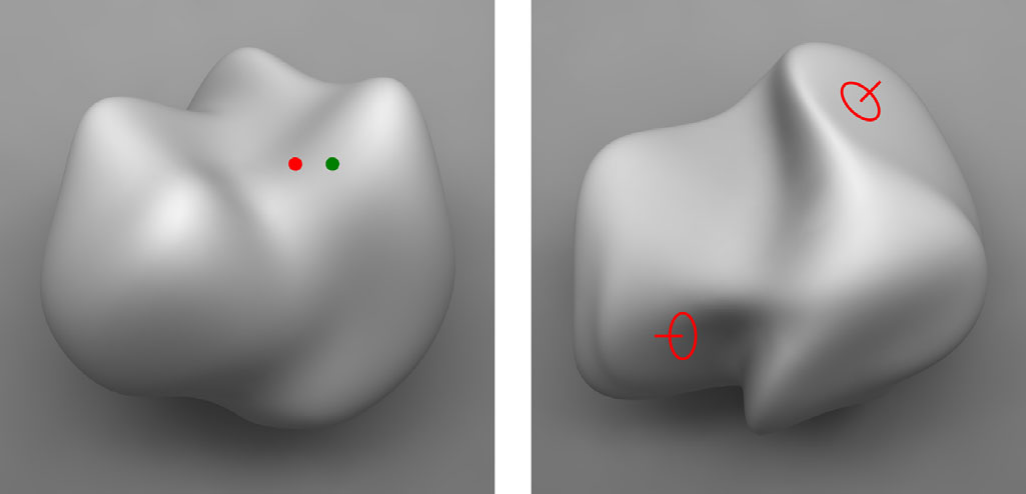
Two local probe tasks to measure the perception of 3D shape on curved surfaces. For the relative depth probe depicted on the left, observers must choose whether the red or green dot appears closer in depth. For the orientation probe on the right, observers must adjust a circular gauge figure until it appears to rest in the tangent plane within a designated local region. Note that the figure on the upper right appears to satisfy that criterion, but the one on the lower left does not.

Figure 14 shows two probe tasks that have been used in numerous psychophysical experiments on the perception of 3D shape. The left panel shows a relative depth probe developed originally by Todd and Reichel (1989), in which observers must judge which of two probe dots appears closer in depth. The right panel shows a relative orientation probe developed by Koenderink, van Doorn, and Kappers (1992), in which observers must adjust the 3D orientation of a circular disk so that it appears to be parallel to the tangent plane of a local surface region. Both of these tasks can be performed very quickly with a high degree of confidence, and it is possible in both cases to compute a 3D surface that is most consistent with the overall pattern of judgments over a large number of probe locations (Koenderink, van Doorn, & Kappers, 1992, 1996; Koenderink, van Doorn, Kappers, & Todd, 2001).

The surfaces computed from either method are highly correlated with one another, and they also have a high degree of test-retest reliability, with *R*^2^ values typically in excess of 0.97 (Egan & Todd, 2015; Todd, Norman, Koenderink, & Kappers, 1997). However, the results also show that the best-fitting surfaces are typically distorted relative to the ground truth by an affine stretching or shearing transformation in depth (e.g., see Figure 3), which is consistent with the known ambiguities of various sources of visual information such as shading or motion (Belhumeur, Kriegman, & Yuille, 1997; Koenderink & van Doorn, 1991; Todd & Bressan, 1990). These findings are also in agreement with the results obtained using global shape adjustment tasks (Bradshaw, Parton, & Glennerster, 2000; Johnston, 1991; Todd & Norman, 2003) or depth magnitude estimations (Loomis & Philbeck, 1999; Todd & Norman, 1991).

The ease and reliability of local orientation and ordinal depth judgments suggest strongly that they are measuring some basic aspect of observers’ perceptual knowledge, but are the structures they reveal a valid representation of 3D shape? The problem with local property maps is that they fail Bunge's second criterion. A set of local positions is not a pure measure of shape because it also encodes the location, orientation, and size of an object. Similarly, it does not provide an easy way to compare two surfaces if they are at different positions and/or orientations. It provides no intuitions about why some types of shape change are more difficult to detect than others, nor does it provide an easy method of subdividing a surface into parts (e.g., hills and valleys). It is especially interesting to note in this regard that Gibson (1979) explicitly disavowed his earlier advocacy of local property maps from the 1950s. He eventually recognized that convexities and concavities are not made up of elementary impressions of slant but are instead unitary features of the layout of surfaces in the environment.

### Contour graphs

#### Pizlo's model

One way of representing 3D shapes with graphs has been proposed by Pizlo, Li, Sawada, and Steinman (2014). They have developed an interesting new algorithm for computing the 3D structures of symmetrical polyhedra from line drawings depicted under orthographic projection, and they have attempted to market that algorithm as a general theory of 3D shape perception (see also Pizlo, 2008). For purposes of the present discussion, our primary interest concerns the specific data structure generated by this analysis (Li, Pizlo, & Steinman, 2009; Li et al., 2011). It consists of a set of 3D Cartesian coordinates for each vertex and a connection graph that describes which vertices are connected to which others (see also Erdogan & Jacobs, 2017). Pizlo et al. (2010) have argued that this model is also applicable to smoothly curved surfaces. However, the data structure in that case is formally equivalent to a depth map.

Thus, Pizlo's analysis shares all the same problems described above for local property maps. First, it is not perceptually intuitive. No one would ever describe a shape colloquially by listing the Cartesian coordinates of its vertices. It does not provide an obvious method for testing if two shapes are the same, even though they might have different sizes and are viewed from different positions and orientations in space. It provides no intuitions about why some types of shape change are more difficult to detect than others. Why, for example, is a change in aspect ratio harder to see than the addition of a bump on a surface? It also does not provide any insight about how an object might be subdivided into namable parts.

#### Edge and vertex labeling

**Figure 15.**
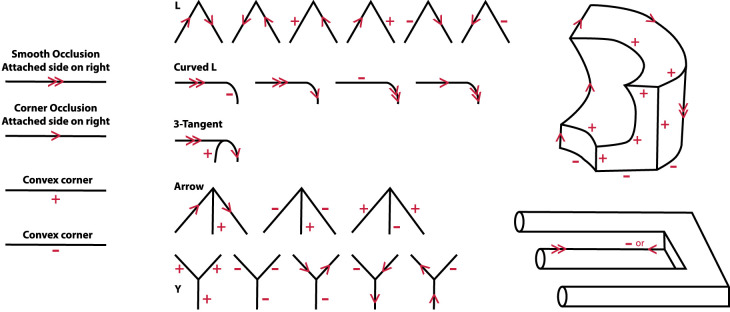
The edge labeling model of Malik (1987). The left panel shows the symbols used for representing different types of edges. The middle panel shows some different types of vertices used by the model and their possible interpretations. The upper right panel shows a complex object with a consistent pattern of labels for all of its edges. The lower right panel shows an impossible object for which some of the edges have no interpretations that are consistent for both of their connected vertices.

Graph data structures have been popular in the field of computer vision since its inception in the 1960s, especially in research on the interpretation of line drawings (Figure 15). One of the first contributions in this field was provided by Guzman (1968). He recognized that vertices on polyhedra can be classified based on the patterns with which their edges coterminate with one another (see also Clowes, 1971; Huffman, 1971, 1977; Malik, 1987; Waltz, 1975). He also recognized that the vertices on each end of an edge can be used to classify whether it is a corner (where a surface is visible on both sides of the edge) or an occlusion (where the surface is only visible on one side). The basic idea behind this analysis is that the connecting edges for each vertex type have a limited set of possible interpretations and that the interpretation of any given edge must be consistent for both of its connected vertices. In some cases, that latter constraint cannot be satisfied, which produces the appearance of an impossible figure. Figure 15 shows the edge labeling model of Malik (1987) with a possible object in the upper right panel and an impossible object in the lower right panel.

It is important to note that the graph representations used in these analyses can be encoded much more efficiently than depth or orientation maps. They are also much more abstract than the one proposed by Pizlo. They are mostly concerned with the topology of polyhedra rather than metric structure, and they are largely invariant over projective transformations and changes in viewing direction. These models make it relatively easy to compare shapes based on the types of vertices they contain and the topology of their graph structures. They can also be used to subdivide objects into distinct parts (see Waltz, 1975). The vertices in these analyses are part of a more general class of visual landmarks that are referred to as singularities, and these structures play a critical role in many other representations of shape that will be described in subsequent sections.

**Figure 16.**
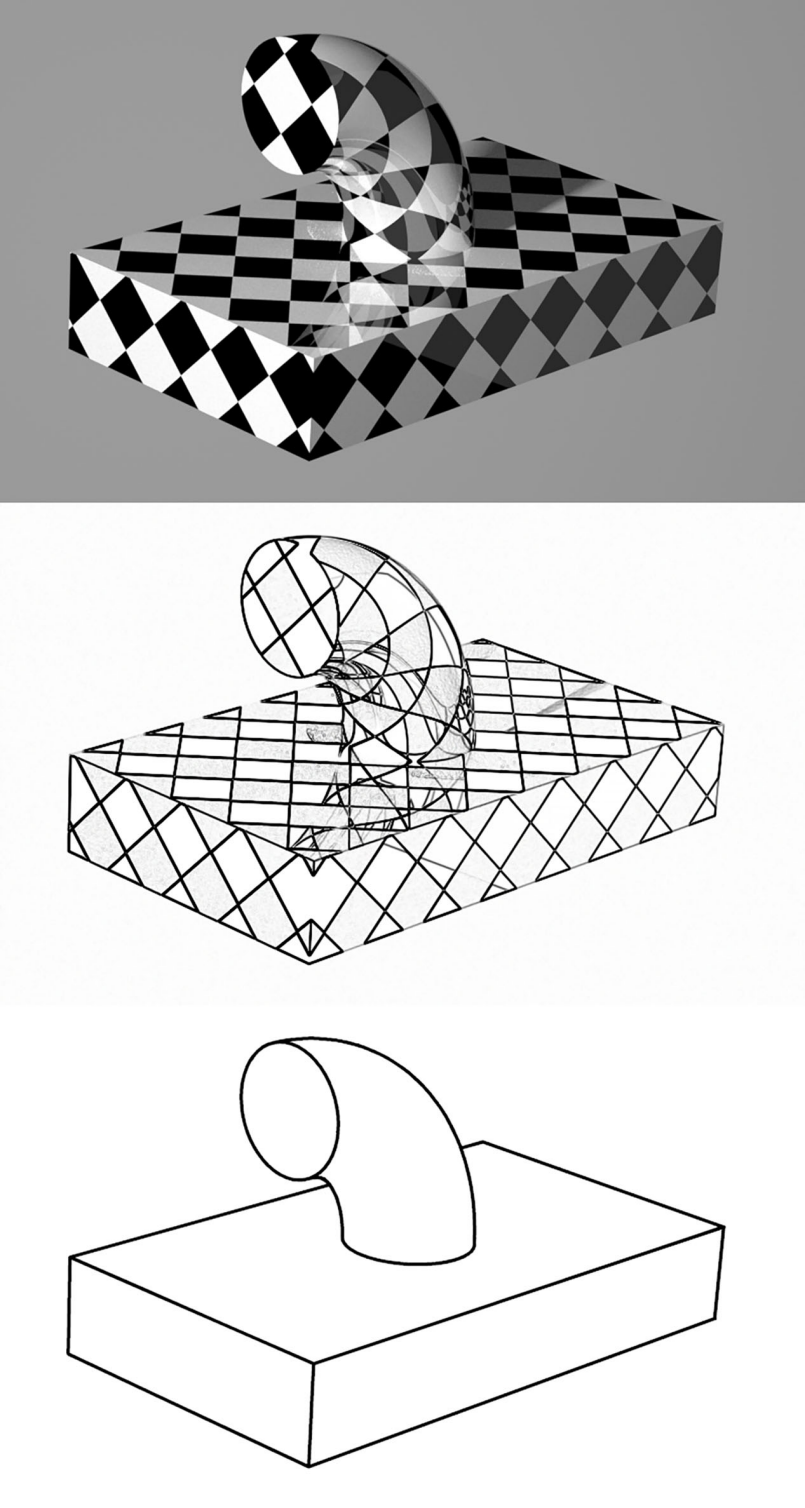
The top panel shows the image of a textured object, with cast shadows and specular reflections. The center panel shows an edge filtered version of that image, and the bottom panel shows a line drawing with only the corners and occlusions.

These traditional models of edge and vertex labeling were designed to be used with line drawings of objects in which all of the lines correspond to occlusions or sharp corners between two faces, and they work quite well in that context. There were also attempts to extend these analyses to natural images of objects, but that is where the research in this area hit a roadblock because of two problems. First, the corners and occlusions in natural scenes are not always well delineated within patterns of shading. Second, the edges in natural scenes can also be caused by other factors, such as specular highlights and abrupt changes of surface reflectance or illumination (e.g., shadows). To demonstrate this more clearly, a shaded image of a textured object with shadows and specular reflections is shown in the top panel of Figure 16. The middle panel shows an edge filtered version of this image, and the bottom panel shows an idealized line drawing with only the corners and occlusions. Human observers can identify the different types of edges quite easily within the pattern of shading, but there are no existing theoretical explanations of how that might be possible.

### Graphs of volumetric parts

#### Generalized cylinders

**Figure 17.**
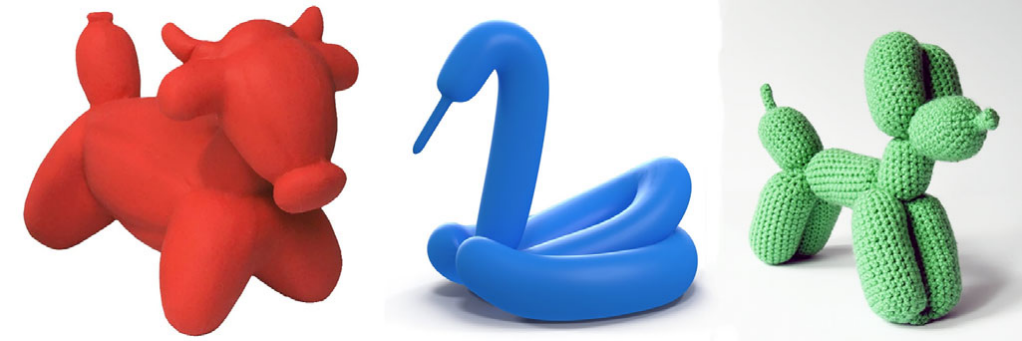
Three objects constructed with cylindrical parts. Note that they are easily recognizable as a bull, a swan, and a dog.

As the field of computer vision started to take off in the 1970s, researchers began to focus on shape representations that can be encoded with relatively few parameters. They were willing to sacrifice the precise details of Euclidean metric structure if they could obtain a more economical description that captures the qualitative aspects of shape. One of the first attempts to achieve that goal was presented by Binford (1971). Inspired by Blum's (1967) medial axis transform, he argued that parts of objects can be modeled as generalized cylinders, which are generated by sweeping a planar curve, such as a circle, along an axis or a spine. Objects that form from natural growth and many manufactured objects tend to have such shapes. This representation is quite similar to a medial axis graph except the axes are surrounded by tubes. It is also possible to allow the sweeping curve to change in size, orientation, or planar shape as it is swept along the spine (Terzopoulos, Witkin, & Kass, 1987, 1988). Figure 17 shows easily recognizable examples of a bull, a swan, and a dog that were created using cylindrical parts.

Generalized cylinders are particularly popular for 3D modeling and character animation in computer graphics. By manipulating the parts separately from one another, it is possible to simulate globally nonrigid motions, such as human gaits, while preserving the local rigidity of each individual limb. By sacrificing metric structure, generalized cylinders can be encoded much more efficiently than local property maps. Nevertheless, because they capture the part structure of objects, they are easy to recognize by human observers.

#### Biederman's model

**Figure 18.**
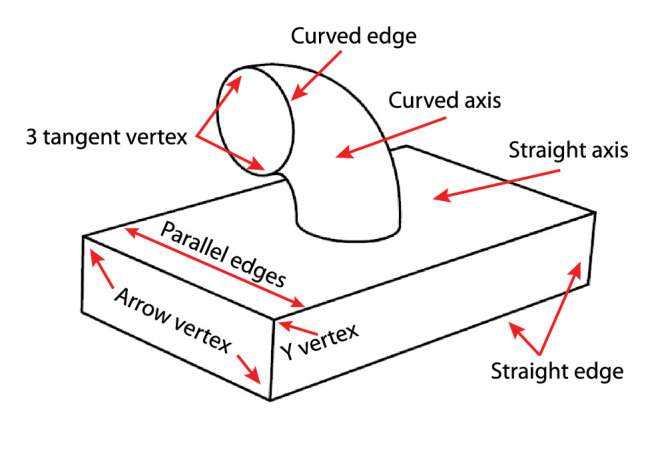
A simple two-part object composed of a brick and a curved cylinder. The brick geon is defined by its parallel straight edges, its straight central axis, its three arrow vertices, and its one Y-vertex. The curved cylinder is defined by its curved central axis and edges and by its two three-tangent vertices.

A more psychologically motivated volumetric representation was proposed by Biederman (1987). He combined a number of the concepts that had been developed earlier by researchers in computer vision, including the vertex classification scheme of Guzman (1968), the part structure decomposition of Binford (1971), and the nonaccidental properties of Witkin and Tenenbaum (1983). He was particularly influenced by the observation that people tend to describe complex novel objects by listing their parts and how they are arranged with respect to one another. The parts in his model include 36 basic shapes such as cylinders, bricks, or bananas that he refers to as geons. These are defined by their vertex types and nonaccidental properties (see Figure 18). Objects are defined by how these elementary parts are arranged with respect to one another. Thus, the underlying data structure of this theory is a graph of graphs. Parts allow a much more efficient coding of shape, because they can be treated as symbolic tokens within higher-order representations.

Biederman's model satisfies all of the criteria for a good measure of shape that we described earlier. It is objective. It is mostly invariant under projective transformations, and it is closely matched to the way that human observers tend to describe complex shapes in terms of parts. It makes it easy to compare shapes simply by listing their respective geons. It explicitly predicts that changes to nonaccidental properties should be easier to detect than changes in metric properties, and it provides a simple procedure for subdividing an object into parts. It is also supported by a wealth of psychophysical data (see Biederman, 1995, for a review).

Nevertheless, there are a couple of limitations of the model that deserve to be highlighted. One of these is that it does not provide a way of identifying the relevant vertex types and nonaccidental properties in natural images. This is probably of little consequence in evaluating it as a psychological model because it is clear that human observers can identify these features quite easily (see Figure 16). However, the absence of a specific algorithm for extracting these features limits the model's potential applications in computer vision.

A second limitation of Biederman's model is that it does not work well with smooth surfaces, like the objects shown in Figure 14, that do not have sharp corners or polyhedral vertices. For example, how is it possible to describe the shape of a tooth, a pattern of draped cloth, or a familiar landscape? The geons described by Biederman are ill suited to represent those objects. Fortunately, there are other techniques for describing smoothly curved objects that will be considered below.

### Patch graphs on 3D surfaces

**Figure 19.**
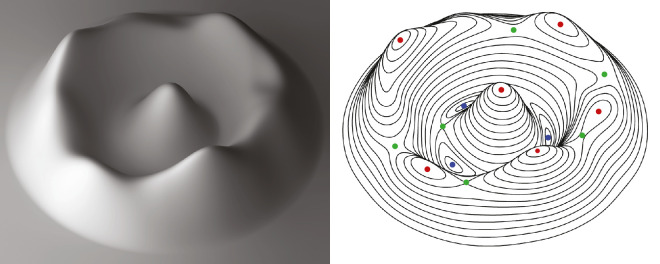
The left panel shows a shaded image of a smoothly curved surface without any sharp corners or a closed boundary contour. Observers typically describe this as a circular ridge with many small bumps and saddles along its crest and a larger bump in the center. The right panel shows the same surface with a series of iso-height contours. The red, green, and blue dots mark height maxima, saddle points, and height minima, respectively.

The top row of Figure 19 shows two depictions of a smoothly curved 3D surface. The one on the left is depicted with shading, and the one on the right is depicted using a pattern of iso-height contours that are also referred to as level sets. Note that this surface has no outer contour from which a medial axis could be constructed, and it has no visible edges or vertices that would allow the application of Biederman's model. Pizlo (2008) has argued that an object like this has no shape because it is asymmetrical, but that claim is clearly ridiculous. If observers are asked to describe its shape, they readily do so in terms of its topographic features. For example, it might be described as a circular ridge with many small bumps and saddles along its crest and a larger bump in the center. This type of object could easily be represented using a depth or orientation map, but how could we do so in a manner that captures the topographic parts that are identified by human observers?

#### Morse graphs of surface extrema

One possible strategy was described 40 years ago in an influential article by Koenderink and van Doorn (1976) on “Singularities of the Visual Mapping.” They described how the Morse theory of differential topology can be used to create a graph of a surface's topography. Consider a continuous function that defines some attribute such as height at each point on a surface. There are three types of local singularities defined by that function: local maxima, local minima, and saddle points. Local maxima and minima of a height map are easily identified where the circular iso-contours converge to a single point. Saddle points occur where the iso-contours intersect one another. For example, the height maxima in the right panel of Figure 19 are marked by small red dots. The height minima are marked by small blue dots, and the height saddle points are marked by small green dots. These singularities define a graph of a surface's topological structure. The local maxima, minima, and saddle points define the nodes of the graph, and the edges are formed by connections between maxima and adjacent saddle points, as well as the connections between saddle points and adjacent minima. This results in a cellular graph structure of local surface patches that captures the qualitative aspects of a surface, without any information about its metric structure. A particularly clear discussion of Morse theory and its application to vision can be found in Kunsberg and Zucker (in press). They use a more advanced version of that theory called the Morse–Smale complex to identify bumps on a surface and to create a graph of its topographic features.

There is some empirical evidence to indicate that local extrema of depth may provide critical information for human perception. For example, Todd and Reichel (1989) found that local relative depth judgments are significantly less accurate (and slower) if the two points to be compared are separated by a depth extremum (see also Koenderink & van Doorn, 1995; Koenderink, van Doorn, & Wagemans, 2015). They argued that these judgments may be based on an ordinal representation, in which regions with a common slope are anchored by local depth minima and maxima. Todd, Oomes, Koenderink, and Kappers (2004) showed that observers are quite accurate at marking local depth extrema along surface scan lines (see also Todd & Thaler, 2010). Although they sometimes mistake inflexion points for local minima, they always add an additional local maximum when that occurs, so that the number of maxima on a scan line is always one greater than the number of minima.

#### Graphs of intrinsic curvature

**Figure 20.**
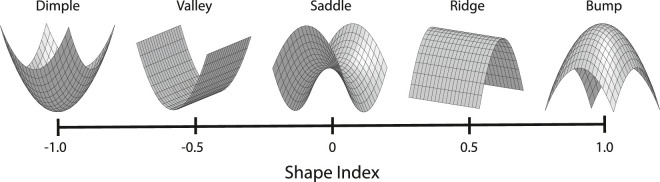
The shape index proposed by Koenderink (1990) partitions local surface regions into five distinct categories that are intuitively identified as bumps, ridges, saddles, valleys, and dimples.

Another possible graph representation of smoothly curved surfaces has been proposed by Koenderink (1990). At any point on a smooth surface, there are two principal directions of curvature that are always orthogonal to one another: one where the curvature (Κ_max_) is larger than in any other direction and another where the curvature (Κ_min_) is smaller than in any other direction. Koenderink noted that Κ_max_ and Κ_min_ can be transformed into two alternative measures: one called curvedness, which varies with scale, and another called the shape index, which is scale invariant (see Figure 20). The shape index partitions surface patches into five qualitatively distinct types: bumps, ridges, saddles, valleys, and dimples. These can define the nodes of a graph, and the adjacency relations between regions define the edges. These different types of curvature are easily identified in the iso-height contours of Figure 19. For bumps and dimples, the contours form closed loops, and for saddles, they diverge away from each other. One important advantage of this approach over the Morse theory analysis is that the shape index is invariant over variations in surface orientation, whereas local extrema of height, depth, or slant are not.

There have been several psychophysical studies that highlight the relevance of these ideas for the visual perception of 3D shape (Lappin, Norman, & Phillips, 2011). For example, research has shown that observers can discriminate variations of the shape index on quadric surface patches (Mamassian, Kersten, & Knill, 1996; Phillips & Todd, 1996; van Damme, Oosterhoff, & van de Grind (1994) and accurately categorize those patches as bumps, ridges, saddles, valleys, or dimples (de Vries, Koenderink, & Kappers, 1993; van Damme & van de Grind, 1993). In addition, Perotti, Todd, Lappin, and Phillips (1998) have shown that observers are much more sensitive to qualitative changes in shape index from motion than they are to quantitative changes in curvedness.

An interesting property of patch graphs of either local singularities or intrinsic curvature is that they are often tightly coupled with sources of visual information, such as shading, texture, motion parallax, and binocular disparity. Note that these image properties all form continuous fields, with the same mathematical structure as a surface. Thus, they all have their own local singularities and patterns of curvature. The patch graphs for motion parallax and binocular disparity are almost identical to those of the visible surfaces from which they arise. That is not the case for shading or texture, but there are still interesting correspondences to examine. For example, Koenderink and van Doorn (1980) have shown that saddle points in the shading field only occur on surface points where one of the principal curvatures is zero.

It is worth noting that patch graphs satisfy all of the criteria discussed earlier for evaluating possible representations of shape. They naturally decompose surfaces into parts, which are closely aligned with how observers describe topographic features on surfaces. They make it easy to compare surface shapes (as long as the number of patches is relatively small), and they also make clear how changes in the topological structure of a graph should be easier to detect than metric changes that do not alter its topology.

### Rims and other extremal curves

**Figure 21.**
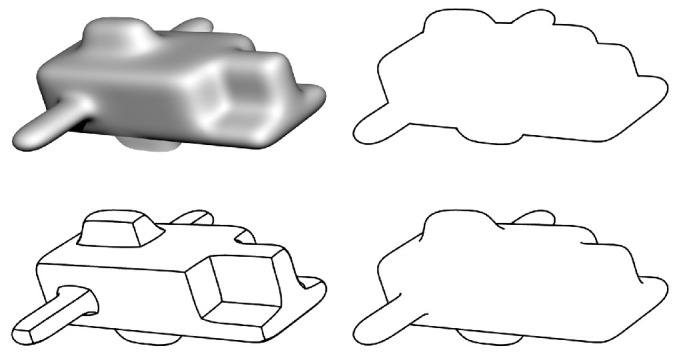
A shaded image of a curved surface and three types of contour drawings. Moving clockwise from the upper left, the panels depict a smoothly shaded image of the object, its silhouette, its rim, and the rim combined with curvature extremal contours, for which K_max_ is a local maximum or K_min_ is a local minimum. Note that the curvature extremal contours dramatically improve quality of the drawings.

What patch graphs cannot do is provide a convenient method for depicting curved surfaces in line drawings. We know that skilled artists are able to create realistic depictions of smoothly curved objects with a relatively small number of image contours, but it is still an open question about where those contours should be placed in order to provide the most perceptually effective depiction. One important contour on a smoothly curved object is its rim (see Figure 21). The rim is defined as the locus of surface points for which the angle between the surface normal and the direction of view is exactly 90°. It is important to note that the rim is different from the silhouette of an object. The silhouette is a subset of the rim that defines the boundary between figure and ground. All points on one side are part of the object, whereas all points on the other side are part of the background. The rim, in contrast, can extend into the interior of a surface or even be disconnected from the silhouette.

Koenderink and van Doorn (1976) performed the first rigorous analysis of how rims can be structured and how they are especially relevant to 3D form perception. They began by describing the different types of singularities that can occur on the rim. That work was later expanded by Damon, Giblin, and Haslinger (2009, 2011, 2016), who were able to enumerate all possible singularities and classify them by their relative stability over small changes in viewing direction. Koenderink (1984a) also presented a theorem that relates the apparent curvature of the rim to the intrinsic curvature of the surface in its immediate local neighborhood (on the attached side). The basic idea is quite simple. Because the curvature perpendicular to the rim is always convex, the curvature parallel to the rim uniquely specifies the qualitative shape index. He also noted that an abrupt termination of the rim can only occur in saddle shaped regions and that artists often draw that incorrectly (see also Koenderink & van Doorn, 1982).

It should be noted in Figure 21 that the rim by itself does not provide a truly compelling impression of the depicted 3D shape. In order to augment that, Phillips, Todd, Koenderink, and Kappers (2003) have argued that additional information can be provided by curvature extremal contours, which connect points where K_max_ is a local maximum or K_min_ is a local minimum. This is implicit in line drawings of polyhedra. The edges have infinite curvature, and the faces have no curvature at all. Of course this is impossible for real objects, but it is a reasonable approximation in many contexts. It is possible to generalize the concept of a sharp edge on polyhedral surfaces to include curvature extremal contours on more rounded surfaces, and the addition of those contours can dramatically enhance pictorial depictions. The lower left panel of Figure 21 shows the rim contours of the shaded image in the upper left panel together with its curvature extremal contours. Note how this produces a much more compelling depiction of the object's shape than what is obtained when the rim contours are presented in isolation.

**Figure 22.**
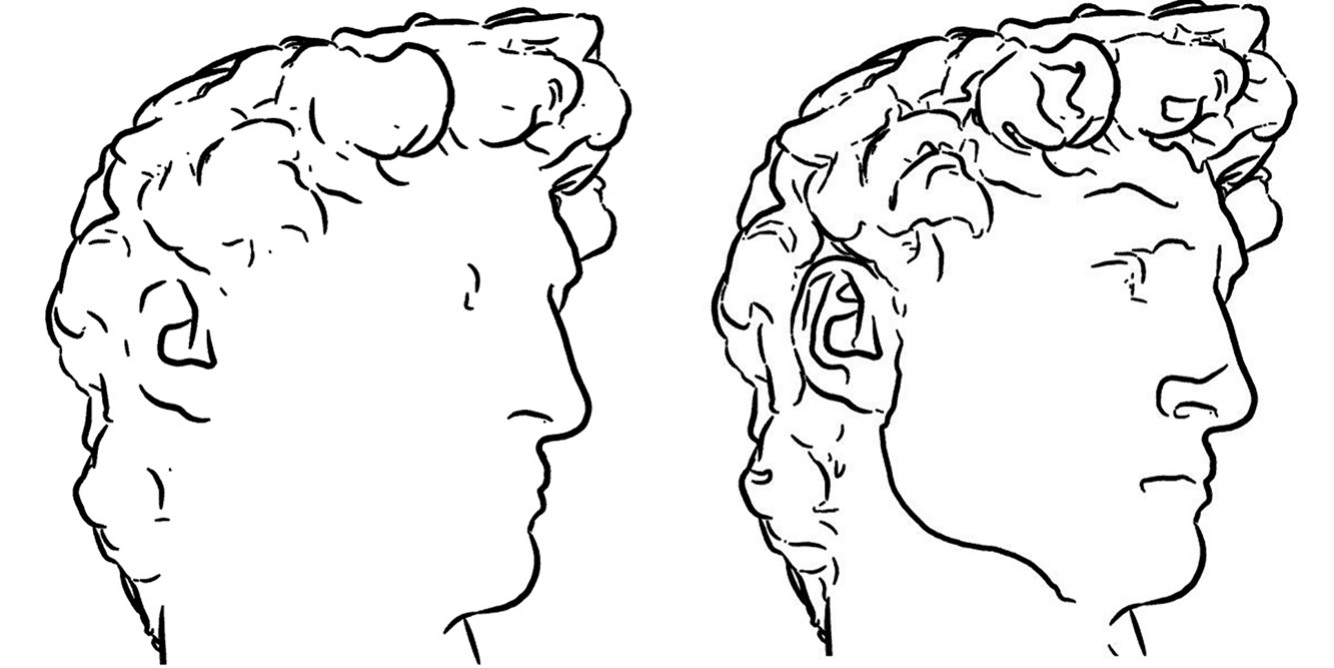
Two line drawings of the head of “David” by Michelangelo (1504) created with different types of contours. The one on the left shows only the rim contours, whereas the one on the right also includes suggestive contours. (Reprinted from DeCarlo, Finkelstein, Rusinkiewicz & Santella, 2003, with permission from the authors.)

Another technique for enhancing the appearance of line drawings has been developed by DeCarlo, Finkelstein, Rusinkiewicz, and Santella (2003). They proposed broadening the definition of the rim somewhat to include points on a surface where the angle between the surface normal and the direction of view is a local maximum that need not be exactly 90°. The locus of these local slant maxima defines what they call suggestive contours. Figure 22 shows a drawing of David with only the rim contours on the left and suggestive contours on the right. Clearly, the one on the right provides a more compelling impression of 3D shape.

### Aspect graphs

**Figure 23.**
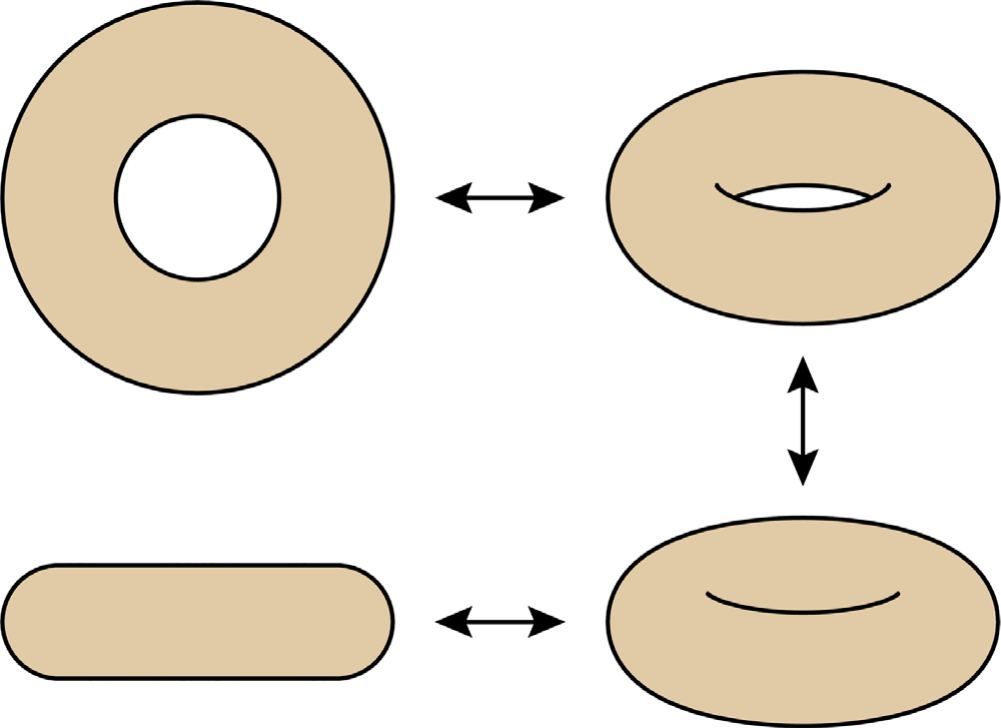
An aspect graph that shows the four possible characteristic views of a torus and the possible transitions between them.

The seminal articles of Koenderink and van Doorn (1976, 1979) did much more than just list the different types of singularities that can occur on the rim. They also described how the structure of the rim changes as a function of the viewing direction. As an object is rotated continuously in depth relative to the observer, singularities on the rim can appear and disappear but always in pairs. Each distinct set of visible singularities is referred to as a characteristic view or an aspect, and there is also a set of possible transitions for how one aspect can be replaced by another. When considered in combination, these structures form a graph, in which the aspects are the nodes, and the transitions between aspects define the edges (see Figure 23).

Damon, Giblin, and Haslinger (2009, 2011, 2016) have further developed these insights within an area of mathematics called singularity theory, and they have also considered the singularities on the borders of cast shadows. In principle, it would be possible to perform similar analyses on a wide range of image structures, such as specular highlights, and extremal contours, but the cost in terms of greater complexity would be quite high.

**Figure 24.**
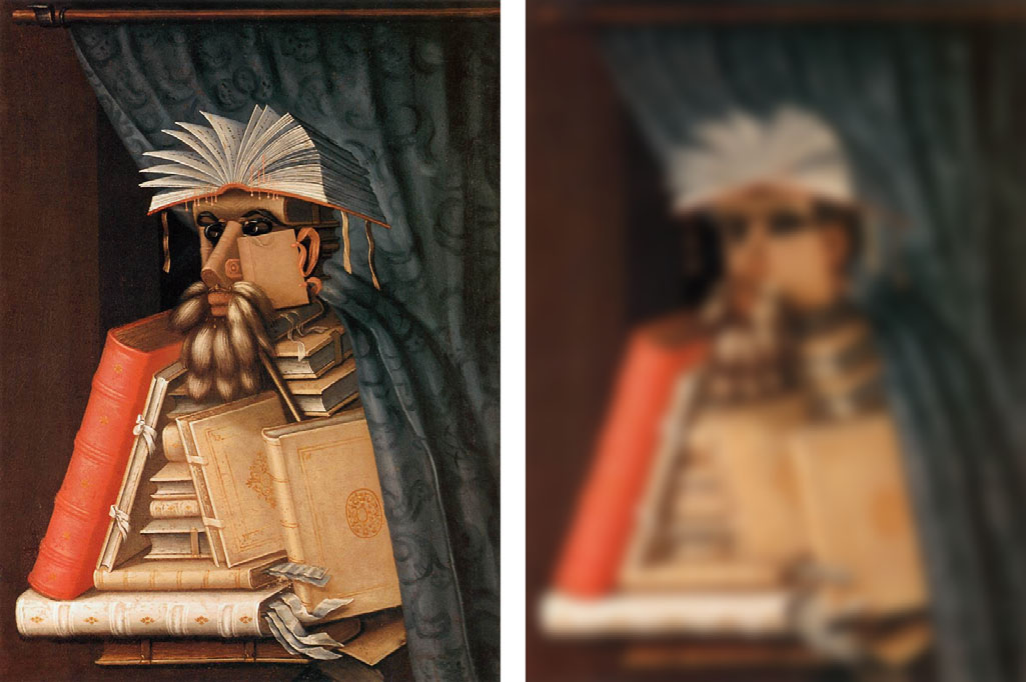
The left panel shows *The Librarian* by Giuseppe Arcimboldo (1566), and the right panel shows a blurred version of it. Note that the original painting can be perceived as a person or an arrangement of books, but when the image is blurred, it can only be perceived as a person.

Because aspect graphs can be extraordinarily complex, it is likely that their relevance to human perception may be limited to relatively simple cases like the torus in Figure 24. One psychophysical investigation to examine this issue was performed by Tarr and Kriegman (2004). They measured observer sensitivity to changes in 3D orientation of a torus and a bell. The results revealed that observers were most sensitive to changes in orientation that resulted in an aspect change, although some aspect transitions had no effect on performance. This is clearly a topic that is deserving of more research.

### Shape as a function of scale

Another complication for the representation of shape is that the appearance of objects can change when they are observed at different distances. For example, consider the shape of a golf ball. At far distances, it appears as a point; at moderate distances, it appears as a sphere; and at very close distances, it appears as an array of hemispherical pits. At distances in between moderate and very close, the spherical structure and the hemispherical pits can be perceived simultaneously, and observers can focus their attentions on either one. The issue of scale is relevant to all of the representations of shape described thus far. For example, the seemingly spurious branches of the Blum medial axis model arise due to the fine-grained structure of the boundary (see left column of Figure 11). The Bayesian analysis of Feldman and Singh (2006) is an effective method of removing those (see right column of Figure 11), but the fine-grained structure is still part of our perceptual experience, especially when parts of a shape are viewed under magnification.

Is it possible to highlight the macroscopic parts in a representation of shape, without discarding the fine-grained structure altogether? Koenderink (1984b) has proposed an elegant method for achieving that goal using a Morse graph of an image, in which the nodes consist of local maxima, minima, and saddle points in the luminance field (see also Lindeberg, 1993; Witkin, 1983). The number of nodes in the graph can be reduced when an image is blurred by the diffusion equation, but it can never increase. Reductions occur when an extremum (i.e., a maximum or a minimum) merges with a saddle point so that both are annihilated. Thus, an image can be defined as a nested set of luminance singularities that vanish in a well-defined sequence with progressive blurring. At some scales, a given singularity will exist as a single blob of light or dark luminance. At courser scales, it may cease to exist at all, and at finer scales, it may be subdivided into additional singularities. The same basic idea can also be applied to height or depth maps of surfaces or even a 2D curve along the boundary of an object. Note that these results are only valid for blurring that is based on the diffusion equation. Other types of blurring can give rise to spurious singularities.

It is clear that human observers are capable of perceiving objects at multiple scales simultaneously. This was first demonstrated in the 16th century by the Italian painter Giuseppe Arcimboldo, who was famous for his imaginative portrait heads made entirely of other objects. For example, the left panel of Figure 24 shows his painting of a librarian that can be perceived as a person or as a collection of books. If the image is blurred as shown in the right panel, then the perception of books is eliminated, but the appearance of a person remains. Similar double images at different scales were also created by Salvador Dali in the 20th century.

A more rigorous psychophysical investigation of multiscale shape perception was performed by Brown and Burbeck (1999). They used the relative orientation probe task shown in Figure 15 on an image of a bumpy sphere. The new twist they added was to vary the size of the probe disk. When the probe disk was small, observers’ judgments were correlated with the local orientations of the individual bumps. However, as the size of the disk was made larger, the observers’ adjustments were more closely associated with the macroscopic spherical shape of the object.

It is obviously not possible to represent all possible scales of an object down to a molecular or subatomic level, which means that no possible representation of shape can be veridical in an absolute sense, but perhaps it is desirable to include a range of scales that are likely to arise in natural vision. This is another interesting area of shape perception that is worthy of additional research.

## Conclusions

One of the things that stands out in this review of possible shape representations is their remarkable variety. Different researchers have tried to capture the concept of shape using many different data structures, ranging from the local property maps of early Gibson and Marr to much more abstract conceptions like medial axes or aspect graphs. Despite the obvious differences among these approaches, there are also some important general principles that bind them together.

### Invariance over change

One important theme that emerges from this discussion is that the concept of shape involves many different object attributes, some of which are more perceptually salient than others (see also Green, 2017; Koenderink, 1990). This suggests that there may not be a single definition of shape that is wholly satisfactory, because different sets of attributes may be relevant in different contexts. For example, the metric properties of Euclidean geometry may be of paramount importance to a tool and die maker but not so much to a biologist who is trying to classify the biological forms of different species.

Within this set of possible shape attributes, we have argued that observers are most sensitive to those that have the greatest stability over change, as was first suggested by Chen (1982). One obvious reason for this is that patterns of visual stimulation are subject to projective distortions, which do not preserve many of the geometric properties of objects in the environment. However, some properties, like collinearity and coincidence relations, are invariant over projective transformations, and those properties are especially informative for human perception.

Curves and surfaces can be described at varying levels of differential structure, including local positions (zero-order), local orientations (first-order), and local curvature (second-order). There is considerable evidence to suggest that curvature is the most important of these for perception of shape, as suggested long ago by Attneave (1954) and Gibson (1979). This is likely because curvature is invariant over changes in the direction of view, whereas position and orientation are not.

### Singularities of the visual mapping

Another important source of information about shape is provided by singular points or curves within patterns of visual stimulation. Singularities occur at local extrema along any continuous function of surface attributes. This could include local extrema of depth or orientation, but they most commonly involve local extrema of curvature. These structures are ubiquitous within many theoretical analyses of shape that have been proposed for both human and machine vision. For example, the edges and vertices of polygons and polyhedra are defined by discontinuities of curvature. The classification of singularities was critical to the early development of computer vision that focused on the interpretation of line drawings, and it remains an active topic of research in both computer vision and mathematics.

Singularities facilitate the perception of shape in two different ways: First, they provide salient landmarks on surfaces that make it possible to establish point-to-point correspondence relations between different objects. When performing correspondence matching tasks, observers consciously seek out salient landmarks to help them triangulate corresponding surface positions (Phillips, Todd, Koenderink, & Kappers, 1997). Second, singularities can also be used to constrain the possible interpretations of a scene in their immediate local neighborhoods and to help classify the contours in an image (Malik, 1987).

### The importance of parts

We have also emphasized the part structure of objects, as first suggested by Blum (1967) and Binford (1971). Parts allow a much more efficient coding of shape because they can be treated as symbolic tokens within higher-order representations. In addition, parts can simplify the computational processing of global shape, by decomposing the analysis into a series of subproblems. The concept of parts does not exist in classical geometry, but it arises frequently in observers’ verbal descriptions of shape. Thus, in order to account for that, a perceptually valid representation of shape should allow a natural decomposition of an object into parts. It is interesting to note that most of the representations proposed in the literature are able to satisfy that criterion. Examples include Biederman's theory of recognition by components, Blum's medial axis transform, and surface patch graphs.

### Possible shape data structures

There are two popular data structures employed in the literature for representing shapes, which we have referred to as maps and graphs. Maps are created by subdividing an object into a dense network of small local neighborhoods, each of which is characterized by some set of attributes. To represent a 2D curve, for example, each local neighborhood could be characterized by its 2D position coordinates. To represent a 3D surface, each local neighborhood could be characterized by its 3D position coordinates or by its surface depth gradient. Depending on the scale of the neighborhoods, maps can encode any possible aspect of surface geometry including the positions and orientations of objects, as well as their shapes. However, it is impossible to separate those properties without additional analyses.

Graphs provide a more abstract type of data structure that can be tailored more specifically to the representation of shape, and because of their hierarchal structure, they provide a natural way for decomposing objects into parts. In general, graphs can be encoded much more efficiently than maps. Moreover, because their nodes and edges typically represent viewpoint-invariant features, they can also provide a plausible explanation of why some shape changes are more difficult to detect than others.

### What is shape?

It is likely that some readers will be unconvinced by this discussion and will insist that the definition of shape must be focused entirely on metric properties. If shape perception were based on anything less than that, they will argue, then our judgments of objects would not be “veridical,” but our day-to-day experiences in the natural environment would seem to contradict that conclusion. Moreover, we know how to compute metric structure from various sources of visual information, so why would the visual system not exploit that capability?

This type of argument is misleading at best. First, it ignores the fact that observers’ judgments of Euclidean metric structure have been studied extensively in the literature on human perception, and with very few exceptions, most of these studies have shown that judgments of metric structure produce large systematic errors and are highly unreliable (see Todd & Norman, 2003, for a review). Second, although there are known algorithms for computing metric structure from visual information in certain contexts, they typically rely on assumptions that are seldom if ever satisfied in the natural environment. For example, they might assume that an object is viewed under orthographic projection (Ullman, 1979) or that it is bilaterally symmetric (Sinha, Ramnath, & Szeliski, 2012). Sometimes these algorithms allow for a family of possible interpretations, and a unique solution is obtained by adopting a minimization procedure, such as picking the one with the smallest volume or surface area (Pizlo, Li, Sawada, & Steinman, 2014). However, whenever those constraints are violated, as is often the case in natural vision, these algorithms will produce systematic errors.

So what is the alternative? Perhaps it is best to think about shape from the functionalist philosophical perspective of James Gibson in his analysis of ecological optics. The function of shape perception in our day-to-day experiences is to discriminate objects and to classify them into distinct categories. It is important to keep in mind that there are an infinity of object properties that could potentially be associated with the concept of shape. One way of determining which ones are most relevant in any given context is to weight them in terms of how reliably they can be measured and how efficiently they can be encoded.

Nonaccidental properties are weighted more heavily than metric properties because they are invariant under optical projection and are therefore more reliable sources of information. Singularities are weighted more heavily than other features because they are so easily recognizable in visual images. Singularities of curvature are weighted most heavily because they are invariant over changes in position and orientation. Similarly, parts are useful in visual perception because they dramatically increase the efficiency of encoding and processing shapes. Graphs are preferred over maps for exactly the same reason, especially when the nodes of those graphs are singularities in image structure.

There are several advantages of defining shape as a weighted configuration of properties, as opposed to the classical definition that shape is what is left over after the effects of position, orientation, and size have been normalized (e.g., Dryden & Mardia, 2016). The weighted property model can allow for crude judgments of relative lengths in different directions (i.e., metric structure) when that is necessary, such as trying to determine if a couch will fit through a doorway. However, it also allows for the categorization of shapes based on the subset of properties they share in common and the categorization of transformations based on the specific properties that are changed and those that remain invariant. This can also explain how we are able to look at sets of objects and immediately discern that their shapes are similar in some ways but different in others (e.g., see Figures 8^9^–10).

**Figure 25.**
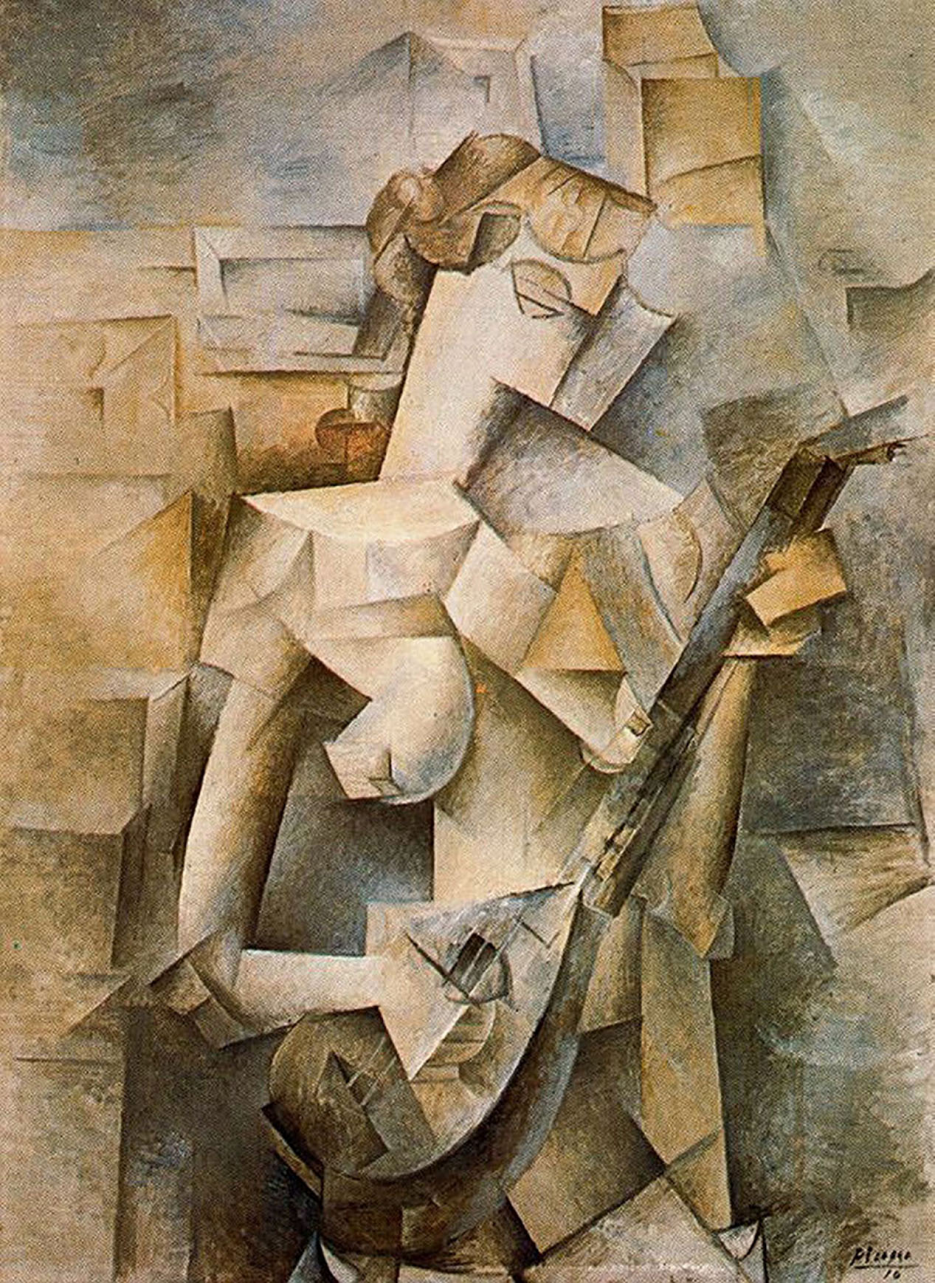
*Girl* *With a Mandolin* by Pablo Picasso (1910). Note how the subject is easily recognizable despite the large amounts of distortion.

This ability is demonstrated most clearly by postimpressionist and cubist painters of the late 19th and early 20th centuries, such as Van Gogh, Cézanne, and Picasso. They began to systematically distort their subjects in an effort to explore the outer limits of representational art. For example, Figure 25 shows a cubist painting of a girl with a mandolin created by Pablo Picasso in 1910. Although this scene is hugely distorted, it is still possible to perceive the overall shape and identity of the subject. How observers are able to recognize scenes with that level of distortion remains a mystery over 100 years later.
